# Association of systemic immune-inflammation-index with all-cause and cause-specific mortality among type 2 diabetes: a cohort study base on population

**DOI:** 10.1007/s12020-023-03587-1

**Published:** 2023-12-04

**Authors:** Chan Yang, Qiangfei Yang, Ziyan Xie, Xi Peng, Hanyu Liu, Chunguang Xie

**Affiliations:** 1 State Key Laboratory of Biotherapy, West China Hospital, TCM Regulating Metabolic Diseases Key Laboratory of Sichuan Province, Chengdu, 610041 Sichuan China; 2 Jianyang City People’s Hospital, Chengdu, 610040 Sichuan China; 3 Hospital of Chengdu University of Traditional Chinese Medicine, TCM Regulating Metabolic Diseases Key Laboratory of Sichuan Province, Chengdu, 610037 Sichuan China

**Keywords:** NHANES, All-cause mortality, Cardiovascular mortality, Prospective cohort study, Population-based study, Systemic immune-inflammation index

## Abstract

**Purpose:**

There have been limited studies examining the prospective association between the Systemic Immune-Inflammation Index (SII), a novel inflammatory marker, and mortality among individuals with diabetes in the United States.

**Methods:**

We utilized data from the National Health and Nutrition Examination Survey (NHANES), a representative sample of US adults, linked with information from the National Death Index.

**Results:**

Our study included 8697 individuals from NHANES spanning the years 1999 to 2018. SII was calculated by dividing the platelet count by the neutrophil count and then dividing that result by the lymphocyte count. We employed multivariable Cox proportional hazards regression analysis to investigate the associations between SII levels and all-cause as well as cause-specific mortality, while adjusting for potential confounding factors. SII levels were categorized into quartiles based on the study population distribution. Over a median follow-up period of 94.8 months (with a maximum of 249 months), we observed a total of 2465 all-cause deaths, 853 deaths from cardiovascular causes, 424 deaths from cancer, and 88 deaths related to chronic kidney disease. After adjusting for multiple variables, higher SII levels were significantly and non-linearly associated with an increased risk of all-cause mortality in Quartile 4 (HR 1.74, 95% CI 1.15–2.63, P for trend = 0.043) when Quartile 1 was used as the reference group. Additionally, we identified a linear association between SII and cardiovascular mortality, with a 70% higher risk of cardiovascular mortality in Quartile 4 (HR 1.70, 95% CI 1.18–3.30, P for trend = 0.041) compared to Quartile 1.

**Conclusion:**

Our findings indicate that SII is significantly associated with an elevated risk of all-cause and cardiovascular mortality in US adults with diabetes.

## Introduction

Type 2 diabetes (T2D), a global public health concern, is projected to affect 643 million people by 2030 and a staggering 783 million by 2045 [1]. Individuals with diabetes face a significantly higher risk of both all-cause and cause-specific mortality compared to those without this condition [2]. The pivotal role of healthcare professionals in illness prevention and management cannot be overstated.

Notably, as far back as 1876, Ebstein demonstrated the potential benefits of sodium salicylate in alleviating symptoms of diabetes [3]. Over the past few decades, diabetes and its associated vascular complications have been increasingly recognized as chronic low-grade inflammatory conditions [4–7]. Proinflammatory cytokines, such as tumor necrosis factor (TNF-α), interleukin-6 (IL-6), and nuclear factor B (NF-B), have been implicated in the activation of various intracellular inflammatory signaling pathways, including JNK and IKK, which can lead to glucose and insulin resistance [4, 6, 8]. Moreover, the involvement of inflammasomes like NLRP3, critical components of inflammatory activation, in the production of interleukin-1 beta (IL-1) has been linked to islet-cell mass and function [4, 9, 10]. Emerging evidence suggests that both innate and adaptive immune responses play pivotal roles in diabetes and its complications [4, 11].

On one hand, innate and adaptive immune cells, including lymphocytes, neutrophils, monocytes, dendritic cells, and macrophages, are primary sources of inflammatory factors and are believed to trigger the IL inflammatory system [11, 12]. Conversely, these immune cells have distinct roles in the development of diabetes and atherosclerosis, which are major contributors to cardiovascular disease (CVD) [13, 14]. Recent research indicates that certain inflammatory and immunological indices based on complete blood counts, such as the platelet-lymphocyte ratio (PLR) and neutrophil-lymphocyte ratio (NLR), may hold promise for predicting cardiovascular events in individuals at risk of primary CVD [15–17]. However, these indices only capture a partial picture of the complex immune-inflammatory landscape.

Enter the Systemic Immune-Inflammation Index (SII), a novel inflammation and immune marker first proposed by Hu et al. [18], SII is calculated using platelet, neutrophil, and lymphocyte counts and has emerged as a robust predictor of systemic inflammation severity [19], It offers insights into both local and systemic inflammation processes [20], and has been linked to various health outcomes, including diabetic depression risk [21], prognosis in cancer patients [22], and the prediction of CVD events [16]. However, the relationship between SII and all-cause, as well as cause-specific mortality—such as cardiovascular mortality, cancer mortality, and chronic renal disease mortality—particularly among individuals with diabetes, remains an open question. To address these knowledge gaps, we conducted an investigation into the associations between SII levels and all-cause and cause-specific mortality in a nationally representative sample of adults with diabetes in the United States.

## Materials and methods

### Study population

Our cohort study included participants sourced from the National Health and Nutrition Examination Survey (NHANES), a regular cross-sectional sampling conducted by the National Center for Health Statistics, under the auspices of the Centers for Disease Control and Prevention. NHANES is renowned for its ability to provide a nationally representative sample of the noninstitutionalized US civilian population. Detailed information regarding NHANES’ sampling methodology and data collection techniques is available on its official website.

### Data collection

Our trained medical personnel collected five categories of data from the participants, covering demographics, dietary habits, physical examination findings, and laboratory test results. This robust dataset allows for the comprehensive analysis of various health-related factors.

### Institutional approval

The NHANES study was conducted with the approval of the Institutional Review Board of the National Center for Health Statistics. All participants provided informed written consent at the time of enrollment, ensuring that ethical standards were upheld throughout the study.

### Inclusion criteria

In our study, we included patients with diabetes who were at least 18 years old, adhering to established criteria for Type 2 diabetes (T2D) diagnosis, as outlined by the American Diabetes Association. These criteria encompassed various indicators, including physician diagnosis, HbA1c levels, fasting glucose levels, random blood glucose levels, oral glucose tolerance test (OGTT) results, and diabetes medication or insulin usage.

### Mortality data

We collected mortality data up to December 31, 2019, by linking our cohort database to the National Death Index. This linkage allowed us to obtain comprehensive statistics on the outcomes of interest.

### Study cohort

In summary, out of the initial 10,1316 participant pool, which included 11,082 individuals with diabetes, 1670 self-reported pregnancy, leaving 9412 participants meeting the T2D and non-pregnant criterion. Among these, 8697 participants had completed the full blood test, enabling us to extract the necessary SII data. These 8697 participants formed the final cohort for our SII analysis.

### Study reporting

Our research adhered to the STROBE (Strengthening the Reporting of Observational Studies in Epidemiology) guidelines, ensuring transparent and comprehensive reporting of our cohort study.

### Laboratory test

#### Laboratory processes

Detailed information regarding the laboratory procedures, including the complete blood count, is available on the NHANES website. In brief, the blood specimens collected from participants underwent a standardized process. After collection, the samples were promptly processed and then frozen at −20 °C. Subsequently, these frozen samples were analyzed by the experts at the National Center for Environmental Health.

#### Blood sample collection

The collection of blood samples for laboratory analysis occurred at a specific juncture during the enrollment process of study participants in NHANES. Notably, these samples were drawn after a designated fasting period. This fasting state necessitated that participants abstain from both food and drink for a specified duration before the blood draw.

#### Timing and standardization

The precise timing of blood sample collection within the NHANES assessment was aligned with the recommended fasting duration required for accurate diagnostic measurements. This careful synchronization ensured consistency across participants and different NHANES survey cycles. This uniformity in timing allows for standardized comparisons, thereby enhancing the reliability and validity of the study’s findings.

#### Calculation of SII

To compute the SII, comprehensive data from the complete blood count were utilized. This dataset included crucial components such as peripheral neutrophil, lymphocyte, and platelet counts. The SII is calculated as follows: platelet count multiplied by the neutrophil count, divided by the lymphocyte count.

### Assessment of covariates

#### Assessment of demographic parameters

In our study, we assessed and categorized several essential demographic parameters through participant interviews based on self-report. These included age, gender, race, ethnicity, education levels, and family income-to-poverty ratio.

#### Body mass index (BMI)

BMI was calculated as the individual’s weight in kilograms divided by the square of their height in meters. BMI values were then categorized into three groups: <25, 25–30, or ≥30.

#### Alcohol consumption classification

Participants were categorized into three groups based on their self-reported daily alcohol consumption. Specifically, they were classified as nondrinkers, moderate drinkers, or heavy drinkers. Moderate drinkers were defined as those consuming less than two drinks per day for men and less than one drink per day for women, while heavy drinkers were those consuming two or more drinks per day for men and one or more drinks per day for women [23].

#### Physical activity (PA) assessment

PA was defined as participating in moderate- to vigorous-intensity sports, fitness programs, or recreational activities for more than 10 min per week. Participants who did not engage in such activities for more than 10 min per week were classified as inactive [24]. The assessment of PA was conducted using the Metabolic Equivalent (MET), a widely recognized measure that represents the relative energy metabolism level during various activities.

#### Healthy eating index (HEI) 2015

We used the Healthy Eating Index (HEI) 2015, developed in alignment with the US Dietary Guidelines for Americans (DGA) 2015–2020, to evaluate dietary patterns among participants [25].

#### Health conditions and medication data

Participants provided self-reported information regarding physician-diagnosed hypertension, hypercholesterolemia, and CVD. Trained personnel collected data on drug consumption over the previous 30 days by comparing participants’ supplied information with drug and dietary supplement databases.

#### Diabetes duration

Participants reported the time of their initial diabetes diagnosis, and by considering their age, we ultimately calculated the duration of diabetes, categorizing it into three groups: <3.0 years, 3.0–10.0 years, or >10.0 years.

#### Clinical assessments

At the time of recruitment, various clinical assessments were conducted, including measurements of HbA1c, triglycerides, total cholesterol, high-density lipoprotein cholesterol, low-density lipoprotein cholesterol levels and renal function (serum creatinine levels).

### Assessment of mortality

All-cause mortality encompassed fatalities resulting from any cause. Cardiovascular mortality was identified using codes I00-I09, I11, I13, I20-I51, and I60-I69 in the International Statistical Classification of Diseases and Related Health Problems, Tenth Revision. Cancer mortality was designated by codes C00-C97. Chronic kidney disease (CKD) mortality was defined by codes N00-N07, N17-N19, and N25-N27.

### Statical analysis

Considering the intricacies of the NHANES examination design, our analyses incorporated weighted adjustments, accounting for clustering and stratification. Person-years were computed for each participant, starting from their enrollment date until the date of death or the conclusion of follow-up on December 31, 2019, whichever occurred first. The assigned weights followed NHANES database criteria, with particular utilization of the mobile examination center (MEC) exam weight (WTMEC2YR) for this study.

To explore the baseline SII quartiles, all 8697 individuals were divided into four groups. For normally distributed data, one-way ANOVA was applied, while the Kruskal-Wallis test was used for data with abnormal distributions. Hazard ratios (HRs) and corresponding 95% confidence intervals (CIs) were calculated using multivariable Cox proportional hazards regression models to assess the associations between SII and all-cause mortality as well as cause-specific mortality risks. The assumption of proportional hazards was evaluated using Schoenfeld residuals [26].

Two multivariable models were constructed. Model 1 adjusted for age (continuous, in years), sex (male or female), and self-reported race and ethnicity (Mexican American, non-Hispanic Black, non-Hispanic White, or other Hispanic). Model 2, an extension of Model 1, further incorporated educational level (less than high school, High School Grad/GED or Equivalent, more than college), family income-to-poverty ratio (<1.0, 1.0–3.0, or ≥3.0), BMI (<25.0, 25.0–29.9, or ≥30.0), drinking status (nondrinker, moderate, or heavy), physical activity (inactive or active), smoking status (never smoker, former smoker, or current smoker), HbA1c level (<7.0% or ≥7.0%), HEI 2015 (in quartiles), diagnosed cardiovascular disease (CVD), hyperlipidemia, self-reported hypertension, diabetes medication use (none, oral glucose-lowering medication, only insulin, oral glucose-lowering medication and insulin), creatinine (continuous) and diabetes duration (<3, 3–10, or ≥10.0). Variables with missing data were subjected to multiple imputation.

To explore the nonlinear relationship between SII levels and mortality, restricted cubic spline analysis (RCS) was performed using four knots (5th, 35th, 65th, and 95th percentiles). Extreme SII values (5% and 95%) were excluded to mitigate the potential influence of outliers, and nonlinearity was assessed via the likelihood ratio test. The associations between SII quartiles and mortality were investigated based on the results of the restricted cubic spline analyses, with the main quartile serving as the reference group. Weighted Kaplan–Meier plots were employed to compare SII levels with all-cause and cause-specific mortality.

Further stratified analyses were conducted by age (<60 or ≥60), sex (male or female), BMI (<30.0 or ≥30.0), and HbA1c level (<7.0% or ≥7.0%). The association between these stratified components was assessed using the *P*-value.

Sensitivity analyses were conducted to assess the robustness of our findings. Firstly, individuals who died within the initial 24 months of follow-up were excluded to reduce the potential for reverse causation bias. Secondly, individuals with a history of CVD were excluded from the primary analysis, as were participants with a history of cancer. Additionally, to control for confounding effects, we applied the multiple propensity scores adjusted technique. Sensitivity analyses also included weighted Kaplan-Meier plots and RCS analysis for all values. All statistical analyses were conducted using R 4.2.1, and statistical significance was defined as a 2-sided *P*-value < 0.05. Data evaluation occurred between May 1, 2022, and November 15, 2022.

## Results

Among the 8697 participants with diabetes (aged ≥18 years old), there were 2465 all-cause deaths, 853 cardiovascular deaths, 424 cancer deaths, and 88 chronic kidney disease deaths during a median follow-up period of 94.8 months (maximum 249 months). Baseline characteristics are presented in Table 1, stratified by SII quartiles (quartile 1: <345.0; quartile 2: 345.0–487.5; quartile 3: 487.5–702.6; quartile 4: ≥702.6). On average, participants were 58 years old, with approximately one-third being non-Hispanic White (36.59%), medium family income to poverty ratio (1.0–3.0, 47.02), and half with obesity (BMI ≥ 30, 55.75%). Additionally, 63.35% reported never drinking, 50.13% were never smokers, and 86.21% had been diagnosed with hyperglycemia. Moreover, 74.68% had a history of CVD, 86.3% had no history of cancer, and 63.98% self-reported hypertension. Significant differences were observed among the four SII groups in terms of sex, race and ethnicity, educational level, BMI, drinking status, physical activity, HEI, self-reported cancer, diabetes medication use, lymphocyte number, neutrophil number, and platelet count (*P*_*value*_ < 0.05). Participants with higher SII levels were predominantly female (1110 or 52.75%), non-Hispanic White (993 or 69.39%), non-drinkers (1072 or 65.33%), had lower HEI scores (mean [SE] 49.94), higher neutrophil numbers (quartile 4: 1319 or 62.76%), higher platelet counts (quartile 4: 1086 or 51.01%), and lower lymphocyte numbers (quartile 4: 280 or 13.63%).

**Table 1:** The baseline characteristic of participants by SII Levels Among Adults with Diabetes in NHANES 1999–2018

	Participants, No (%)					
	SII level					
Characteristic	Total	Quartile 1 (17.3–345.0)	Quartile 2 (345.0–487.5)	Quartile 3 (487.5–702.6)	Quartile 4 (702.6–11700.0)	*P* value
Age, years	58.95 (0.19)	59.18 (0.38)	58.85 (0.38)	59.00 (0.38)	58.83 (0.41)	0.91
Sex						0.01
Female	4222 (48.55)	972 (45.27)	1071 (47.97)	1069 (49.08)	1110 (52.75)	
Male	4475 (51.45)	1200 (54.73)	1105 (52.03)	1105 (50.92)	1065 (47.25)	
Race and ethnicity						<0.0001
Mexican American	1765 (20.29)	398 (9.64)	501 (11.07)	453 (8.78)	413 (7.85)	
Non-Hispanic Black	2123 (24.41)	767 (23.33)	512 (13.57)	449 (11.71)	395 (10.29)	
Non-Hispanic White	3182 (36.59)	595 (52.45)	705 (59.56)	889 (65.57)	993 (69.39)	
Other	1627 (18.71)	412 (14.59)	458 (15.81)	383 (13.94)	374 (12.47)	
Educational level						0.002
Less than high school	3248 (37.42)	863 (29.39)	845 (26.11)	772 (22.37)	768 (23.79)	
Equivalent and more than college	3439 (39.62)	820 (45.32)	827 (47.55)	909 (52.91)	883 (50.12)	
Equivalent high school	1994 (22.97)	484 (25.29)	503 (26.34)	489 (24.72)	518 (26.08)	
Family income to poverty ratio						0.11
<1.0	1834 (23.38)	470 (17.90)	453 (16.39)	450 (15.48)	461 (16.20)	
1.0–3.0	3688 (47.02)	932 (44.20)	887 (40.58)	925 (40.48)	944 (43.08)	
≥3.0	2322 (29.6)	549 (37.90)	618 (43.03)	611 (44.04)	544 (40.72)	
BMI_group						0.002
<25.0	1211 (14.46)	295 (13.59)	299 (11.81)	286 (10.63)	331 (13.93)	
25.0–29.9	2494 (29.79)	698 (30.61)	650 (26.73)	599 (27.16)	547 (23.94)	
≥30.0	4668 (55.75)	1122 (55.80)	1157 (61.46)	1200 (62.21)	1189 (62.12)	
Drinking status						0.02
Heavy	1416 (21.82)	389 (26.95)	360 (24.55)	358 (23.37)	309 (19.28)	
Moderate	962 (14.83)	235 (14.70)	249 (16.63)	243 (15.54)	235 (15.39)	
None	4111 (63.35)	953 (58.35)	1047 (58.83)	1039 (61.09)	1072 (65.33)	
Physical activity						<0.0001
Active	3292 (64.8)	871 (68.82)	897 (71.38)	799 (63.97)	725 (60.07)	
Inactive	1788 (35.2)	432 (31.18)	419 (28.62)	452 (36.03)	485 (39.93)	
Smoking status						0.43
Former	2822 (33.61)	661 (31.33)	707 (34.17)	712 (33.81)	742 (35.57)	
Never	4209 (50.13)	1122 (51.74)	1088 (49.82)	1042 (49.64)	957 (46.99)	
Now	1365 (16.26)	331 (16.92)	323 (16.00)	340 (16.55)	371 (17.43)	
HbA1c,%						0.68
<7.0	4775 (56.75)	1208 (59.15)	1178 (57.98)	1183 (60.20)	1206 (60.16)	
≥7.0	3639 (43.25)	910 (40.85)	940 (42.02)	911 (39.80)	878 (39.84)	
HEI 2015	51.11 (0.21)	52.46 (0.43)	51.20 (0.41)	51.09 (0.43)	49.94 (0.41)	<0.001
CVD						0.13
No	6263 (74.68)	1602 (76.22)	1654 (78.78)	1531 (75.21)	1476 (75.12)	
Yes	2123 (25.32)	509 (23.78)	462 (21.22)	557 (24.79)	595 (24.88)	
Self-reported Cancer						0.04
No	7449 (86.3)	1900 (85.67)	1890 (84.77)	1864 (85.76)	1795 (81.80)	
Yes	1183 (13.7)	258 (14.33)	274 (15.23)	290 (14.24)	361 (18.20)	
Hyperlipidemia						0.15
No	1199 (13.79)	338 (13.42)	287 (11.82)	260 (10.93)	314 (13.33)	
Yes	7497 (86.21)	1833 (86.58)	1889 (88.18)	1914 (89.07)	1861 (86.67)	
Diabetic medicine use						<0.0001
only_insulin	640 (11.59)	135 (10.13)	142 (9.04)	152 (10.29)	211 (16.98)	
only_medicine	3021 (54.73)	770 (50.62)	801 (56.73)	730 (54.42)	720 (51.14)	
Insulin+medicine	710 (12.86)	166 (10.70)	183 (12.23)	179 (12.60)	182 (12.50)	
none	1149 (20.82)	345 (28.56)	285 (22.00)	281 (22.68)	238 (19.38)	
Self-reported Hypertension						0.71
No	3029 (36.02)	767 (38.03)	779 (38.58)	776 (36.48)	707 (36.77)	
Yes	5380 (63.98)	1352 (61.97)	1336 (61.42)	1319 (63.52)	1373 (63.23)	
Lymphocyte count 10^3^/ul						<0.0001
Quartile 1 (0.2–1.6)	1864 (21.43)	212 (10.07)	316 (13.48)	461 (19.73)	875 (37.16)	
Quartile 2 (1.6–2.1)	2431 (27.95)	497 (23.33)	625 (28.91)	662 (31.91)	647 (30.93)	
Quartile 3 (2.1–2.6)	2001 (23.01)	529 (23.16)	549 (25.53)	550 (26.14)	373 (18.28)	
Quartile 4 (2.6–45.2)	2401 (27.61)	934 (43.44)	686 (32.09)	501 (22.23)	280 (13.63)	
Neutrophils count,10^3^/ul						<0.0001
Quartile 1 (0.3–3.4)	2139 (24.59)	1294 (55.63)	561 (23.31)	218 (9.18)	66 (3.10)	
Quartile 2 (3.4–4.3)	2101 (24.16)	556 (26.85)	775 (36.12)	539 (24.28)	231 (9.70)	
Quartile 3 (4.3–5.4)	2158 (24.81)	253 (13.40)	586 (27.37)	760 (34.71)	559 (24.44)	
Quartile 4 (5.4–19.4)	2299 (26.43)	69 (4.11)	254 (13.20)	657 (31.82)	1319 (62.76)	
Platelet count,10^3^/ul						<0.0001
Quartile 1 (12–197)	2133 (24.53)	1035 (47.55)	564 (26.94)	332 (16.63)	202 (9.89)	
Quartile 2 (197–236)	2153 (24.76)	609 (28.23)	673 (32.50)	557 (25.21)	314 (14.44)	
Quartile 3 (236–286)	2197 (25.26)	369 (16.80)	581 (25.53)	674 (30.51)	573 (24.66)	
Quartile 4 (286–975)	2214 (25.46)	159 (7.42)	358 (15.03)	611 (27.66)	1086 (51.01)	
Diabetes duration, years						0.012
<3	1256 (14.44)	299 (13.93)	323 (14.86)	319 (14.67)	316 (14.53)	
3–10	3942 (45.33)	981 (45.71)	996 (45.84)	978 (44.99)	987 (45.38)	
>10	3499 (40.23)	866 (40.35)	884 (40.68)	877 (40.34)	872 (40.09)	
Creatinine, mg.dl	0.852 (0.18)	0.789 (0.21)	0.874 (0.13)	0.848 (0.25)	0.895 (0.19)	0.821

**Fig. 1:**
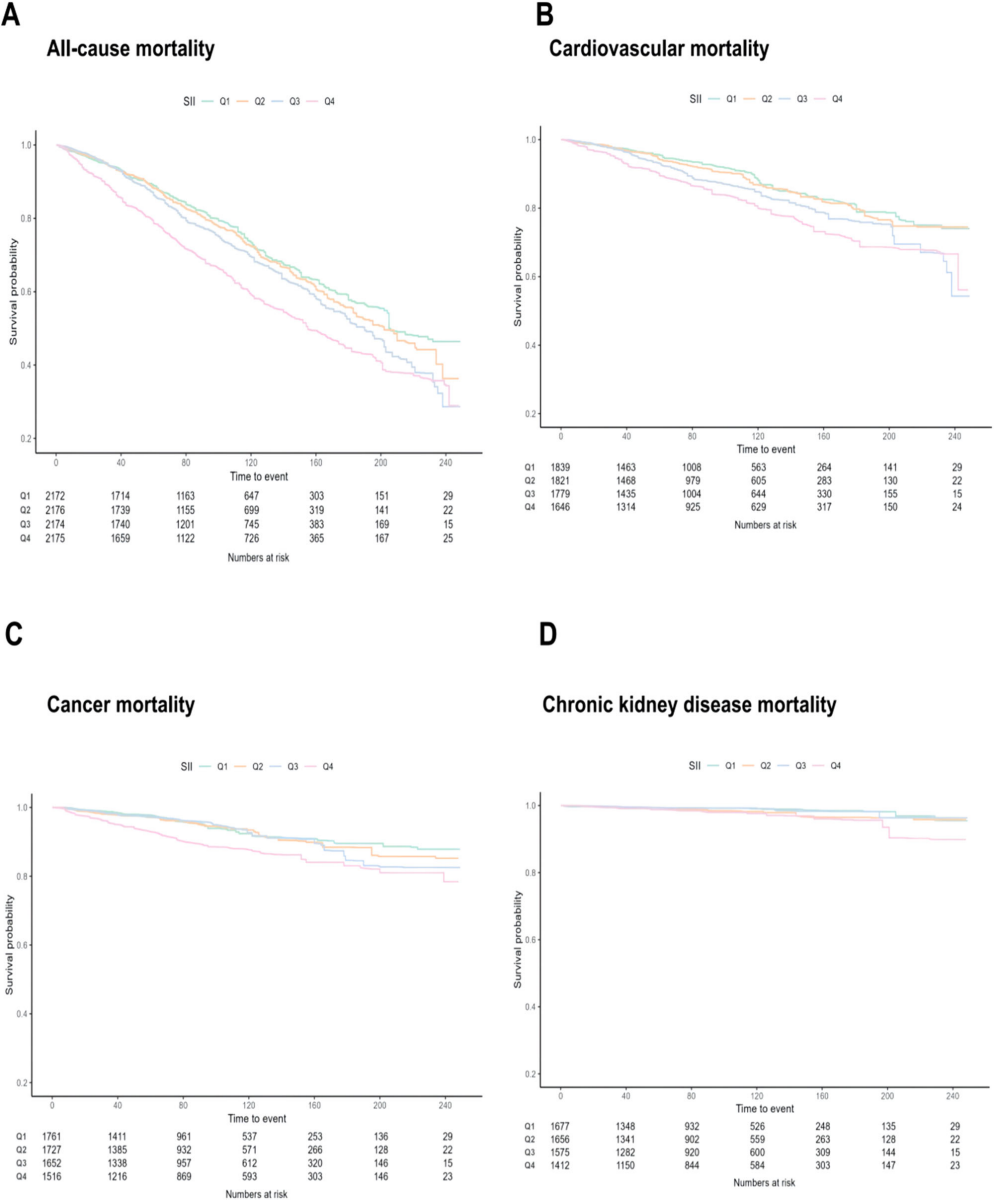
Weighted Kaplan–Meier plots illustrating the association of SII with All-cause Mortality and cause specific among Adults with Diabetes in the National Health and Nutrition Examination Survey (NHANES) 1999–2018. **A** all-cause mortality. **B** cardiovascular mortality. **C** cancer mortality. **D** chronic kidney disease mortality

A total of 2465 all-cause deaths, 853 cardiovascular deaths, 424 cancer deaths, and 88 chronic kidney disease deaths were identified during a median follow-up of 94.8 months (maximum 249 months). Weighted Kaplan-Meier plots of SII levels with all-cause mortality and cause-specific mortality indicate that higher SII levels are associated with lower survival probabilities as follow-up time increases (Fig. 1A–D, *p* < 0.001 for all-cause and cardiovascular mortality; *p* > 0.05 for cancer and chronic kidney disease mortality).

**Fig. 2:**
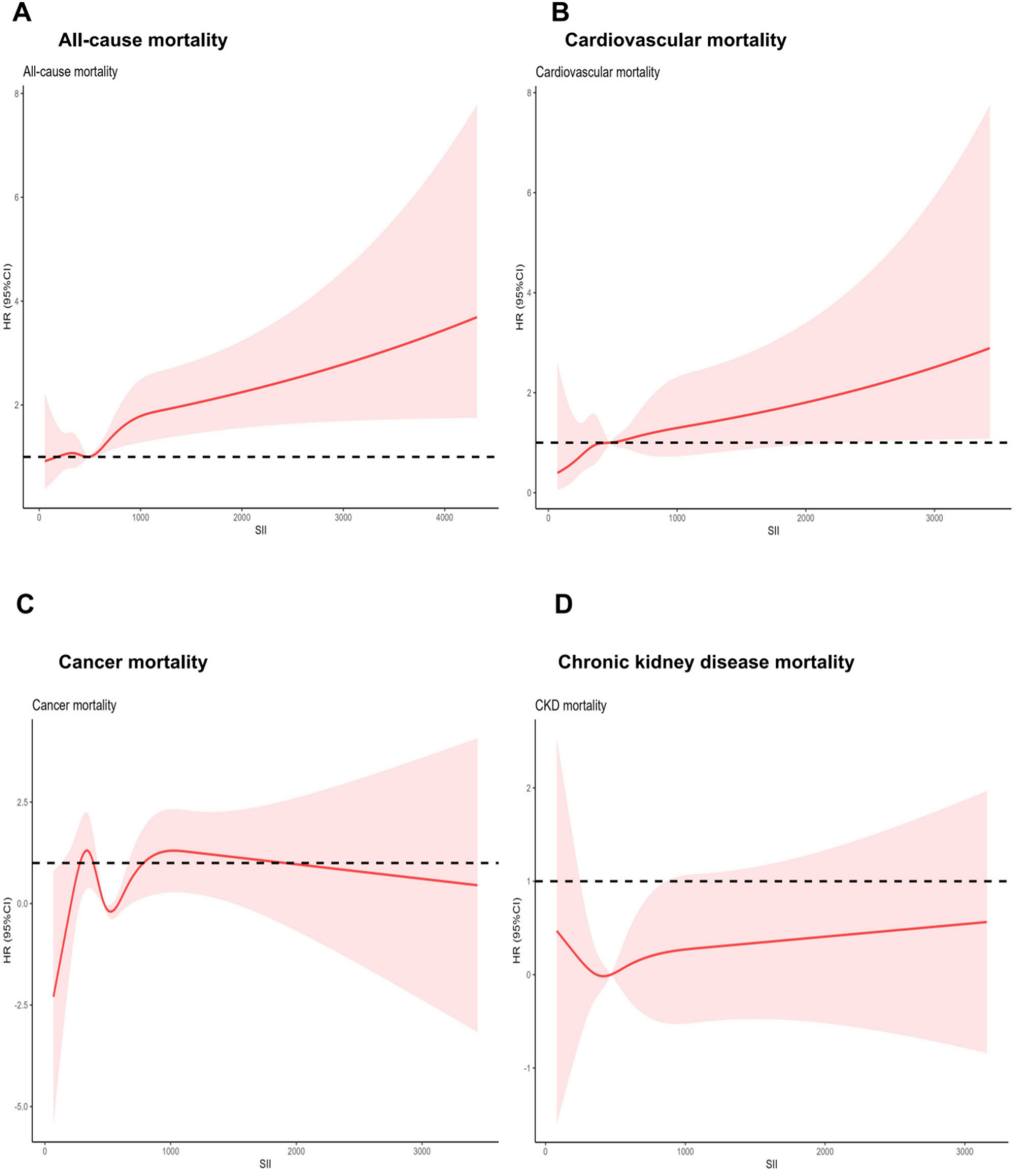
HRs illustrating the association of SII with All-cause Mortality Among Adults and cause specific mortality with Diabetes in the National Health and Nutrition Examination Survey (NHANES) 1999–2018. **A** all-cause mortality. **B** cardiovascular mortality. **C** cancer mortality. **D** chronic kidney disease mortality. Hazard ratios (solid lines) and 95% CIs (shaded areas) were adjusted for age (continuous), sex (male or female), race and ethnicity (self-reported Mexican American, non-Hispanic Black, non-Hispanic White, or other), educational level (<high school, high school or equivalent, or college or above), BMI (<25.0, 25.0–29.9, or ≥30.0), family income-to-poverty ratio (<1.0, 1.0–3.0, or >3.0), drinking status (nondrinker, moderate drinker, or heavy drinker), physical activity (inactive or active), smoking status (never smoker, former smoker, or current smoker), HbA1c (<7% or >7%), HEI (in quartiles),diagnosed CVD (yes or no), self-reported hypertension (yes or no), Hyperlipidemia (yes or no), diabetes medication use (none, oral glucose-lowering medication, only insulin, or oral glucose lowering medication and insulin), Lymphocyte count 103/uL (in quartiles), Neutrophils count 103/uL (in quartiles), Platelet count 103/uL (in quartiles), creatinine (continuous) and diabetes duration (<3, 3–10, or ≥10.0)

In the Cox regression models, after multivariable adjustment, when compared to the reference group (the first quartile), the highest SII levels (the fourth quartile) showed a positive association with HRs for all-cause mortality and cardiovascular mortality (HR 1.74, 95% CI 1.15–2.63, p for trend = 0.043; and HR 1.7, 95% CI 1.18–3.30, p for trend = 0.041; respectively) (Table 2). However, no significant associations were found between each quartile of SII group and cancer mortality and chronic kidney disease mortality (HR 1.36, 95% CI 0.61–3.07, p for trend = 0.637; and HR 0.9, 95% CI 0.11–7.14, p for trend = 0.545; respectively) (Table 2). Further dose-response association analysis through restricted cubic splines showed a U-shaped association between SII and all-cause mortality, and a linear association between SII and cardiovascular mortality (Fig. 2A, B), whereas SII levels showed no significant association with cancer and chronic kidney mortality (Fig. 2C, D).

**Table 2:** Hazard ratios of All-Cause Mortality and Cause specific by SII Levels Among Adults with Diabetes in NHANES 1999–2018.

Model	Hazard ratio (95% CI)				
	Quartile of systemic immune-inflammation index				
	Q1 (17.3–345.0)	Q2 (345.0–487.5)	Q3 (487.5–702.6)	Q4 (702.6–11700.0)	*P*_trend_
All-cause mortality Death,No./total No.	511/2172	546/2176	615/2174	793/2175	
Crude	1(ref)	0.99 (0.84, 1.17)	0.99 (0.84, 1.16)	1.38 (1.18, 1.60)	<0.0001
Model 1	1(ref)	1.00 (0.85, 1.16)	0.97 (0.83, 1.13)	1.44 (1.25, 1.67)	<0.0001
Model 2	1(ref)	1.21 (0.79, 1.85)	1.24 (0.82, 1.87)	1.74 (1.15, 2.63)	0.043
CVD mortality Death,No./total No.	178/1839	191/1821	220/1779	264/1646	
Crude	1(ref)	1.09 (0.84, 1.40)	1 (0.76, 1.31)	1.45 (1.10, 1.91)	0.038
Model 1	1(ref)	1.12 (0.90, 1.39)	1.04 (0.78, 1.38)	1.67 (1.26, 2.21)	0.004
Model 2	1(ref)	1.54 (0.80, 2.95)	2.06 (0.92, 4.52)	1.7 (1.18, 3.30)	0.041
Cancer mortality Death,No./total No.	100/1761	97/1727	93/1652	134/1516	
Crude	1(ref)	0.79 (0.53, 1.18)	0.67 (0.46, 0.97)	0.99 (0.71, 1.39)	0.857
Model 1	1(ref)	0.76 (0.51, 1.15)	0.66 (0.44, 0.98)	1.07 (0.75, 1.53)	0.796
Model 2	1(ref)	1.04 (0.44, 2.44)	0.64 (0.23, 1.79)	1.36 (0.61, 3.07)	0.637
Kidney mortality Death, No./total No.	16/1677	26/1656	16/1575	30/1412	
Crude	1(ref)	1.75 (0.83, 3.66)	0.79 (0.32, 1.93)	1.62 (0.74, 3.52)	0.588
Model 1	1(ref)	1.9 (0.87, 4.15)	0.93 (0.36, 2.41)	2.17 (0.93, 5.04)	0.229
Model 2	1(ref)	1.05 (0.14, 8.05)	0.07 (0.01, 0.52)	0.9 (0.11, 7.14)	0.545

Model 1 was just adjusted for age (continuous), sex (male of female), and race and ethnicity (self-reported Mexican American, non-Hispanic Black, non-Hispanic White, or other). Model 2 was adjusted for model 1+ educational level (< high school, high school or equivalent, or college or above), BMI ( < 25.0, 25.0–29.9, or ≥30.0), family income-to-poverty ratio (3.0), drinking status (nondrinker, moderate drinker, or heavy drinker), physical activity (inactive or active), smoking status (never smoker, former smoker, or current smoker), HbA1c (7%), HEI (in quartiles),diagnosed CVD (yes or no), self-reported hypertension (yes or no), Hyperlipidemia (yes or no), diabetes medication use (none, oral glucose-lowering medication, only insulin, or oral glucose lowering medication and insulin), creatinine (continuous) and diabetes duration (<3, 3–10, or ≥10.0)

In the stratified analyses, no significant interaction were found between SII and these strata variables with the risk of all-cause mortality and cardiovascular mortality after accounting several testes (all *P*_*interaction*_ > 0.05) (Table 3).

**Table 3:** All-Cause Mortality and Cardiovascular mortality by SII Levels Among Adults with Diabetes in NHANES 1999–2018

	Hazard ratio (95% CI) for All-cause mortality				
Characteristic	Q1(17.3–345.0)	Q2(345.0–487.5)	Q3(487.5–702.6)	Q4(702.6–11700.0)	*P*_*interaction*_
Age, years, No./total					0.078
<60 (362/2465)	1[Reference]	1.9 (0.79–4.6)	0.84 (0.33–2.12)	1.52 (1.07–3.23)	
≥60 (2103/2465)	1[Reference]	1.12 (0.7–1.78)	1.4 (0.85–2.32)	1.82 (1.08–3.05)	
Sex					0.513
Female (1071/2465)	1[Reference]	1.13 (0.74–1.72)	1.15 (0.69–1.93)	1.59 (1.09–2.55)	
Male (1394/2465)	1[Reference]	1.71 (0.78–3.76)	1.79 (0.91–3.52)	2.72 (1.33–5.57)	
BMI,kg/m^2^					0.64
<30 (1223/2303)	1[Reference]	0.76 (0.44–1.34)	1.01 (0.57–1.8)	1.38 (1.14–2.57)	
≥30.0 (1080/2303)	1[Reference]	1.51 (0.9–2.53)	1.25 (0.66–2.38)	1.94 (1.17–3.52)	
HbA1c, %					0.483
<7.0 (1299/2296)	1[Reference]	1.03 (0.61–1.75)	1.0 (0.59–1.71)	1.46 (1.09–2.73)	
≥7.0 (997/2296)	1[Reference]	1.36 (0.66–2.8)	1.39 (0.68–2.83)	2.22 (1.13–4.37)	

	Hazard ratio (95% CI) for Cardiovascular mortality				
Characteristic	Q1(17.3–345.0)	Q2(345.0–487.5)	Q3(487.5–702.6)	Q4(702.6–11700.0)	*P*_*interaction*_
Age, years, No./total					0.40
<60 (110/853)	1[Reference]	1.51 (0.38–3.05)	0.77 (0.22–2.73)	1.42 (1.06–2.55)	
≥60 (743/853)	1[Reference]	1.15 (0.51–2.55)	2.65 (1.07–6.58)	1.88 (1.18–3.21)	
Sex					0.375
Female (369/853)	1[Reference]	1.0 (0.43–2.33)	1.44(0.55–3.78)	1.29(1.13–2.05)	
Male (484/853)	1[Reference]	1.38 (0.96–1.93)	2.64 (1.45–4.83)	2.47 (1.77–3.64)	
BMI, kg/m^2^					0.14
<30 (415/787)	1[Reference]	0.6 (0.26–1.41)	1.59 (0.65–3.91)	1.76 (1.19–3.04)	
≥30.0 (372/787)	1[Reference]	1.95 (0.66–3.75)	2.01 (0.56–4.26)	2.62 (1.23–4.69)	
HbA1c, %					0.851
<7.0 (443/785)	1[Reference]	0.91 (0.34–2.44)	1.5 (0.52–4.34)	1.77 (1.09–2.51)	
≥7.0 (342/785)	1[Reference]	1.55 (0.96–4.92)	3.16 (0.81–2.4)	2.47 (1.16–4.03)	

Hazard ratio (95% CIs) was adjusted for age (continuous), sex (male of female), and race and ethnicity (self-reported Mexican American, non-Hispanic Black, non-Hispanic White, or other),educational level (< high school, high school or equivalent, or college or above), BMI ( < 25.0, 25.0–29.9, or ≥30.0), family income-to-poverty ratio (3.0), drinking status (nondrinker, moderate drinker, or heavy drinker), physical activity (inactive or active), smoking status (never smoker, former smoker, or current smoker), HbA1c (7%), HEI (in quartiles),diagnosed CVD (yes or no), self-reported hypertension (yes or no), Hyperlipidemia (yes or no), diabetes medication use (none, oral glucose-lowering medication, only insulin, or oral glucose lowering medication and insulin), creatinine (continuous) and diabetes duration (<3, 3–10, or ≥10.0)

In the sensitivity analyses, the results showing that the SII level had a significant positive association with all-cause mortality remained robust. When excluding individuals who died within 24 months of follow-up, and comparing with the reference group (the first quartile), in the fourth quartile group, the HRs were 1.28 (95% CI, 1.06–1.55) in the crude model, 1.4 (95% CI, 1.16–1.68) in model 1, and 1.55 (95% CI, 1.11–2.66) in model 2 (Supplementary Fig. S1 and Supplementary Table S1). When excluding individuals who had a history of CVD at baseline, and comparing with the reference group (the first quartile), in the fourth quartile group, the HRs were 1.28 (95% CI, 1.09–1.51) in the crude model, 1.36 (95% CI, 1.17–1.58) in model 1, and 1.63 (95% CI, 1.04–2.57) in model 2 (Supplementary Fig. S2 and Supplementary Table S1). When excluding individuals who had a history of cancer at baseline, and comparing with the reference group (the first quartile), in the fourth quartile group, the HRs were 1.35 (95% CI, 1.14–1.60) in the crude model, 1.47 (95% CI, 1.27–1.70) in model 1, and 1.69 (95% CI, 1.05–2.74) in model 2 (Supplementary Fig. S3 and Supplementary Table S1). Moreover, the RCS curve did not change substantially (Supplementary Figs. S4–6). However, all estimated risks between SII and cardiovascular mortality were reduced. After removing individuals who died within 24 months of follow-up, and comparing with the reference group (the first quartile), the highest levels of SII (the fourth quartile) only showed a positive association with the HRs of cardiovascular mortality after full adjustment (model 2, HR 1.7, 95% CI 1.26–2.29, *p* for trend = 0.749) (Supplementary Fig. S7 and Supplementary Table S2). After removing individuals who had a history of CVD at baseline, and comparing with the reference group (the first quartile), the highest levels of SII (the fourth quartile) only showed a positive association with the HRs of cardiovascular mortality after full adjustment (model 2, HR 2.04, 95% CI 1.38–3.03, *p* for trend = 0.312) (Supplementary Fig. S8 and Supplementary Table S2). And after removing individuals who had a history of cancer at baseline, and comparing with the reference group (the first quartile), the highest levels of SII (the fourth quartile) only showed a positive association with the HRs of cardiovascular mortality after full adjustment (model 2, HR 1.59, 95% CI 1.17–2.16, *p* for trend = 0.479) (Supplementary Fig. S9 and Supplementary Table S2). Besides, the RCS curve did not change significantly (Supplementary Figs. S10–12).

## Discussion

Our hypothesis that SII, a predictive marker for cardiovascular disease (CVD) incidence and various cancer prognoses, could be independently linked with all-cause mortality and cause-specific mortality in diabetes turned into a significant journey of discovery. Diabetes, characterized by chronic low-grade inflammation, carries a substantial 2–4-fold higher risk of cause-specific death compared to its non-diabetic counterparts. In this pioneering prospective cohort study, we ventured into uncharted territory, exploring whether SII could be the missing link between inflammation and mortality in diabetes. Our findings are a testament to the depth of this relationship.

To the best of our knowledge, our study marks the first of its kind, unveiling the association between SII and an elevated risk of all-cause and cardiovascular mortality in diabetes. We charted this unexplored territory with rigor, employing a large-scale prospective approach with a nationally representative sample of U.S. adults living with diabetes. Even in the presence of established risk factors such as BMI, Healthy Eating Index (HEI), smoking, drinking, and diabetic medication usage, our results remained resolute. Robustness was a recurring theme as we subjected our findings to a battery of sensitivity and stratified analyses.

This extensive research effort confirms what previous studies hinted at – that SII is linked to an increased risk of CVD events and mortality [16]. This time, we have shone a spotlight on the relationship between SII and all-cause and cardiovascular mortality among individuals with diabetes. The implications are far-reaching, with potential repercussions for how we understand and manage diabetes-related mortality. While our study did also observe a positive association between SII levels and cancer mortality and chronic kidney disease mortality, statistical significance eluded us, which not consistent with the exited evidence that show a positively association between SII levels and cancer mortality [27–29] and chronic kidney disease mortality [30], possibly due to limited case numbers, multifarious covariates, and the quartile-based SII classification. It calls for more extensive prospective studies to corroborate these intriguing findings.

Acknowledging the influence of age and gender on SII levels [15, 31], we thoughtfully stratified our analyses. Remarkably, similar patterns emerged in age groups of 60 and above and among males. The story remained consistent when we delved into BMI and HbA1c subgroups, revealing that the SII effect might be most pronounced in older, male, obese individuals with uncontrolled glucose levels.

While our study illuminates the link between SII and mortality, the mechanisms underlying this association remain enigmatic. Emerging evidence suggests that immunological inflammation plays a pivotal role in both diabetes and CVD [4, 11]. It hints at SII’s potential immunomodulatory effects. Yet, we acknowledge that the exact mechanisms require deeper mechanistic investigations.

The current study’s strengths include its prospective study design, relatively large sample size, and use of a nationally representative sample of diabetes in the United States, allowing us to generalize our findings. Furthermore, because the NHANES collected such detailed data, we were able to control for a wide variety of potentially confounding variables, including socioeconomic status, race/ethnicity, dietary and lifestyle factors, and comorbidities. Some limitations must also be considered. First, due to the nature of the observational research design, our data cannot be utilized to infer causality. Second, we remark that the current study is unable of explaining the putative mechanism of SII and mortality. Third, baseline characteristics may change over time, concealing the underlying relationship between SII and mortality. Fourth, despite correcting for diabetic medicines and HbA1c levels, the severity of diabetes could not be fully accounted for in the current study due to a lack of information. Fifth, because we categorised the SII based on the quartile of the study population, our findings may not be comparable to other studies that employed other cut points. Sixth, residual or unknown confounding cannot be ruled out altogether. Besides, it is conceivable that SGLT2 inhibitors (SGLT2-i) or GLP-1 receptor agonists (GLP-1 RA) could influence cardiovascular mortality in T2D patients, our analysis was limited by the absence of specific identification for SGLT2-i or GLP-1 RA medications in the NHANES database. In future studies, it would be valuable to explore these medications in greater detail, possibly through clinical trials or other data sources that can provide more specific drug information.

## Conclusion

In conclusion, our study adds a significant chapter to the narrative of inflammation, SII, and mortality in diabetes. It underscores the need for further exploration, inviting the scientific community to unravel the complex web of factors linking SII, inflammation, and the ultimate outcome of mortality in diabetes.

### Supplementary information

Supplementary

## Data Availability

The raw data supporting the conclusions of this article will be found in the NHANES website, without undue reservation.
