# Supplementary material for: Association of systemic immune-inflammation-index with all-cause and cause-specific mortality among type 2 diabetes: a cohort study base on population
Source: Endocrine. 2023 Dec 4;84(2):399–411. doi: 10.1007/s12020-023-03587-1 (PMC11076376; doi:10.1007/s12020-023-03587-1)
Supplement: Supplementary file 1 — Supplementary [file 12020_2023_3587_MOESM1_ESM.docx]

**Supplementary**

**Supplementary Figure S1.** Weighted Kaplan-Meier plots explaining the association of SII with All-Cause Mortality Among Adults With T2D excluding Less Than 2 Years of Follow-up.

**Supplementary Figure S2.** Weighted Kaplan-Meier plots explaining the association of SII with All-Cause Mortality Among Adults With T2D excluding a CVD history at the baseline.

**Supplementary Figure S3.** Weighted Kaplan-Meier plots explaining the association of SII with All-Cause Mortality Among Adults With T2D excluding a cancer history at the baseline.

**Supplementary Table S1.** Hazard ratios of All-Cause Mortality by SII Levels Among Adults With T2D Excluding Less Than 2 Years of Follow-up, CVD and cancer at baseline.

**Supplementary Figure S4.** RCS curve Association of SII Levels With All-Cause Mortality Among Adults With T2D excluding Less Than 2 Years of Follow-up.

**Supplementary Figure S5.** RCS curve Association of SII Levels With All-Cause Mortality Among Adults With T2D excluding a CVD history at the baseline.

**Supplementary Figure S6.** RCS curve Association of SII Levels With All-Cause Mortality Among Adults With T2D excluding a cancer history at the baseline.

**Supplementary Figure S7.** Weighted Kaplan-Meier plots explaining the association of SII with Cardiovascular Mortality Among Adults With T2D excluding Less Than 2 Years of Follow-up.

**Supplementary Figure S8.** Weighted Kaplan-Meier plots explaining the association of SII with Cardiovascular Mortality Among Adults With T2D excluding a CVD history at the baseline.

**Supplementary Figure S9.** Weighted Kaplan-Meier plots explaining the association of SII with Cardiovascular Mortality Among Adults With T2D excluding a cancer history at the baseline.

**Supplementary Table S2.** Hazard ratios of Cardiovascular Mortality by SII Levels Among Adults With T2D Excluding Less Than 2 Years of Follow-up, CVD and cancer at baseline.

**Supplementary Figure S10.** RCS curve Association of SII Levels With Cardiovascular Mortality Among Adults With T2D excluding Less Than 2 Years of Follow-up.

**Supplementary Figure S11.** RCS curve Association of SII Levels With Cardiovascular Mortality Among Adults With T2D excluding a CVD history at the baseline.

**Supplementary Figure S12.** RCS curve Association of SII Levels With Cardiovascular Mortality Among Adults With T2D excluding a cancer history at the baseline.


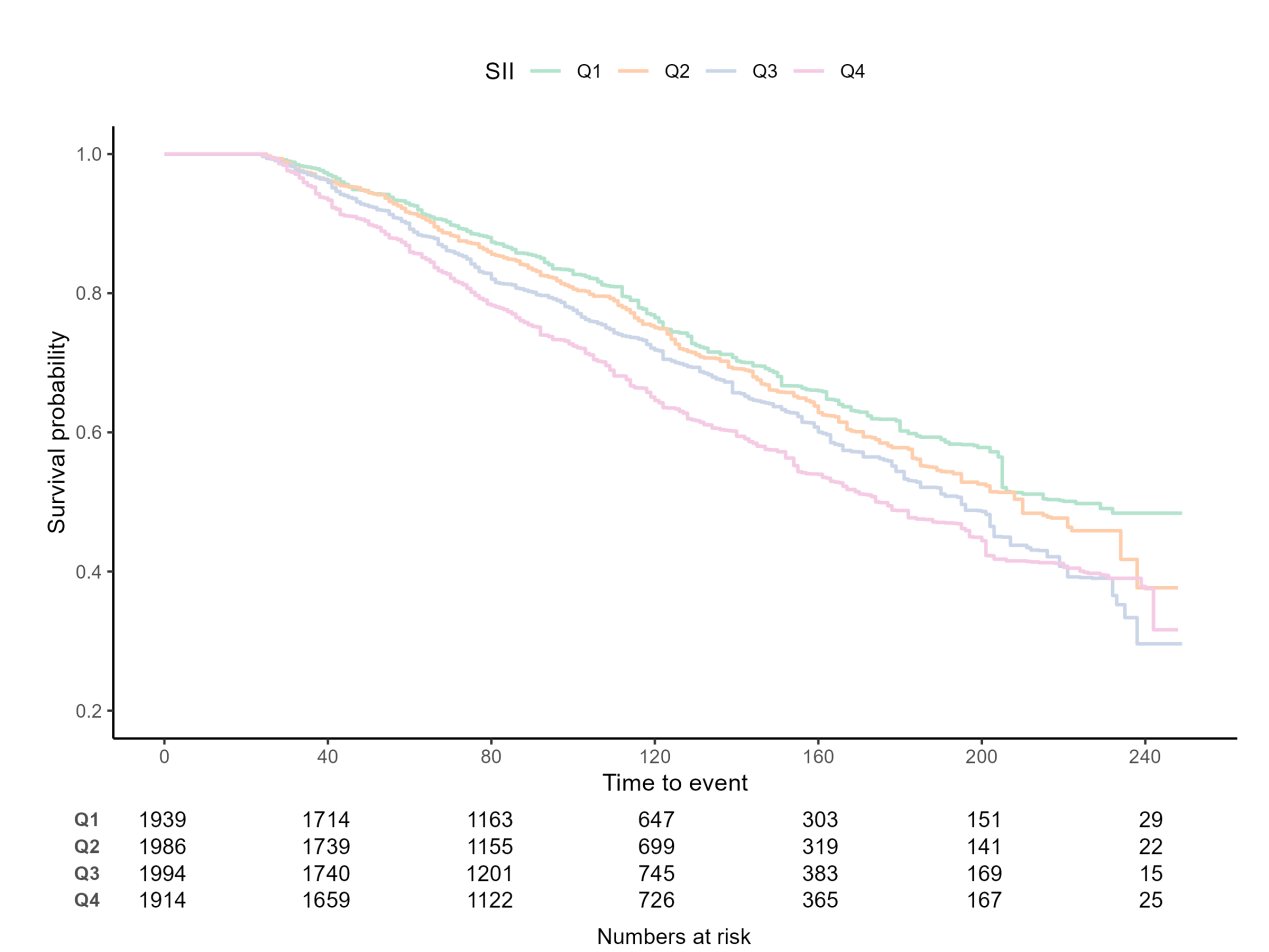


**Supplementary Figure S1.** Weighted Kaplan-Meier plots explaining the association of SII with All-Cause Mortality Among Adults With T2D excluding Less Than 2 Years of Follow-up.


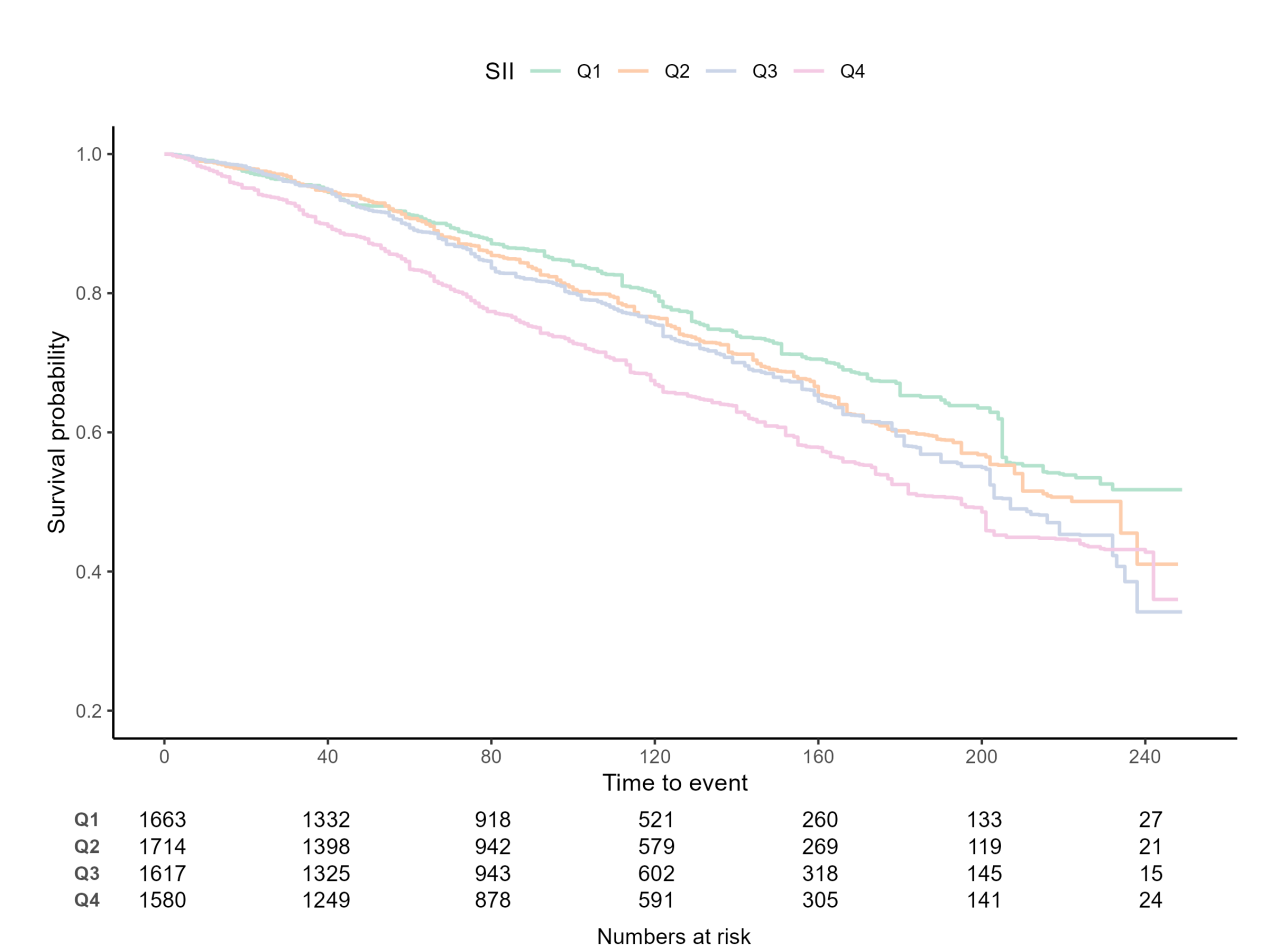


**Supplementary Figure S2.** Weighted Kaplan-Meier plots explaining the association of SII with All-Cause Mortality Among Adults With T2D excluding a CVD history at the baseline.


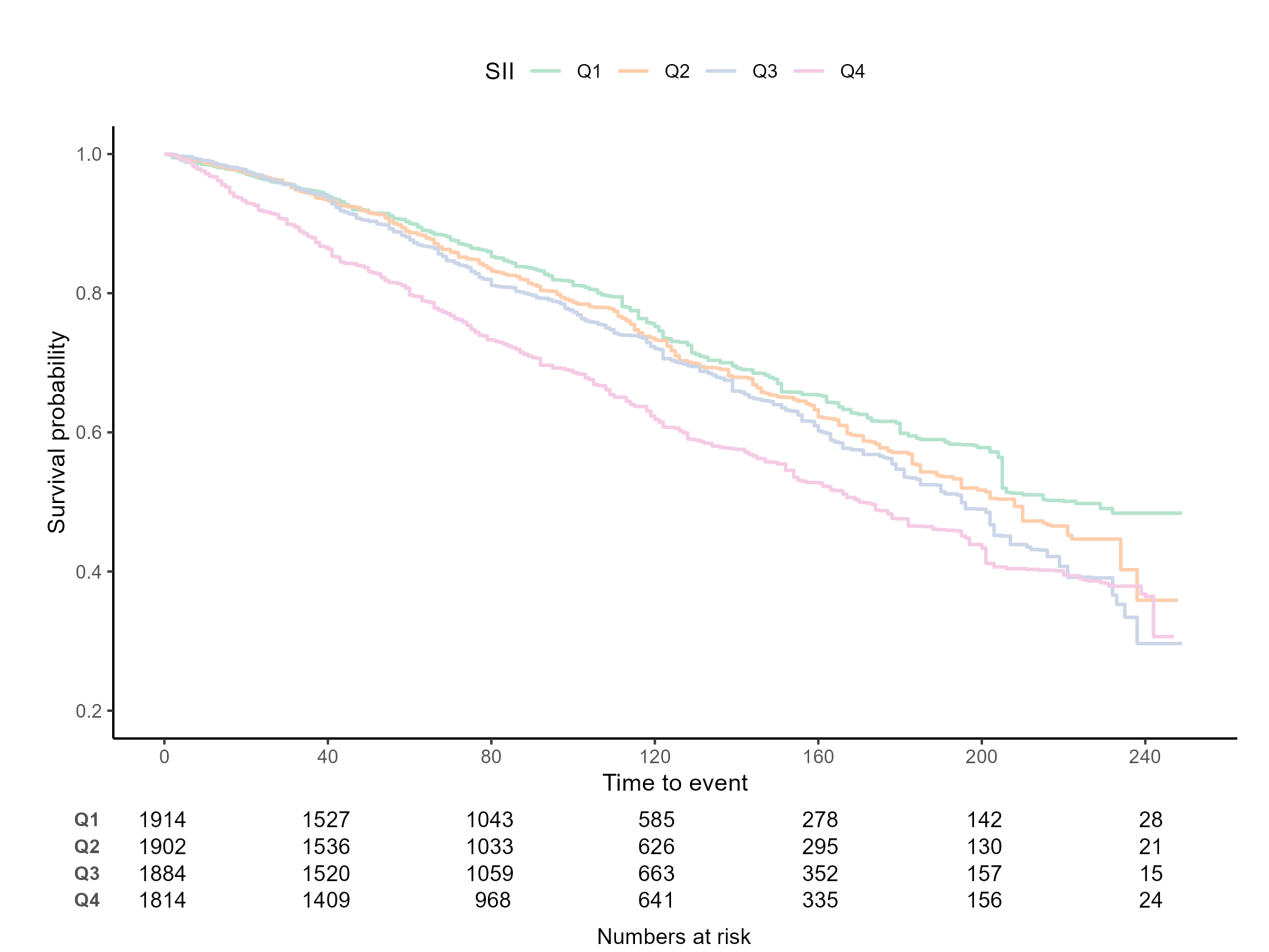


**Supplementary Figure S3.** Weighted Kaplan-Meier plots explaining the association of SII with All-Cause Mortality Among Adults With T2D excluding a cancer history at the baseline.

| Model | **Hazard ratio(95% CI) for all-cause mortality** | | | |  |
| --- | --- | --- | --- | --- | --- |
|  | **Quartile of systemic immune-inflammtion index** | | | |  |
|  | Q1(17.3-345.0) | Q2(345.0-487.5) | Q3(487.5-702.6) | Q4(702.6-11700.0) | P_trend_ |
| Removing less than 24 months follow-up(N=7833) |  |  |  |  |  |
| Crude | 1(ref) | 1.02(0.81, 1.30) | 0.93(0.75, 1.15) | 1.28(1.06, 1.55) | 0.004 |
| Model 1 | 1(ref) | 1.04(0.85, 1.27) | 0.94(0.75, 1.17) | 1.4(1.16, 1.68) | 0.06 |
| Model 2 | 1(ref) | 1.15(0.69, 1.91) | 1.01(0.58, 1.75) | 1.55(1.11, 2.66) | 0.263 |
| Removing CVD at baseline(N=6574) |  |  |  |  |  |
| Crude | 1(ref) | 1.01(0.83, 1.23) | 0.98(0.81, 1.18) | 1.28(1.09, 1.51) | 0.078 |
| Model 1 | 1(ref) | 1(0.84, 1.19) | 0.96(0.79, 1.16) | 1.36(1.17, 1.58) | <0.001 |
| Model 2 | 1(ref) | 1.17(0.78, 1.75) | 1.23(0.80, 1.89) | 1.63(1.04, 2.57) | 0.004 |
| Removing cancer at baseline(N=7514) |  |  |  |  |  |
| Crude | 1(ref) | 1(0.82, 1.21) | 0.98(0.80, 1.20) | 1.35(1.14, 1.60) | <0.001 |
| Model 1 | 1(ref) | 1.01(0.84, 1.20) | 0.99(0.81, 1.21) | 1.47(1.27, 1.70) | <0.001 |
| Model 2 | 1(ref) | 1.2(0.77, 1.87) | 1.23(0.79, 1.92) | 1.69(1.05, 2.74) | <0.001 |

**Supplementary Table S1.** Hazard ratios of All-Cause Mortality by SII Levels Among Adults With T2D Excluding Less Than 2 Years of Follow-up, CVD and cancer at baseline. Hazard ratios (95% CI) were adjusted for educational level (less than high school, High School Grad/GED or Equivalent, more than college, family income to poverty ratio (<1.0, 1.0-3.0, or ≥3.0), BMI (<25.0, 25.0-29.9, or ≥30.0), drinking status (nondrinker, moderate, or heavy), physical activity (inactive or active), smoking status (never smoker, former smoker, or current smoker), HbA1c level (<7.0% or ≥7.0%), HEI 2015 (in quartile), diagnosed cardiovascular disease (CVD), hyperlipidemia, self-reported hypertension, and diabetes medication use (none, oral glucose-lowering medication, only insulin, oral glucose lowering medication and insulin).


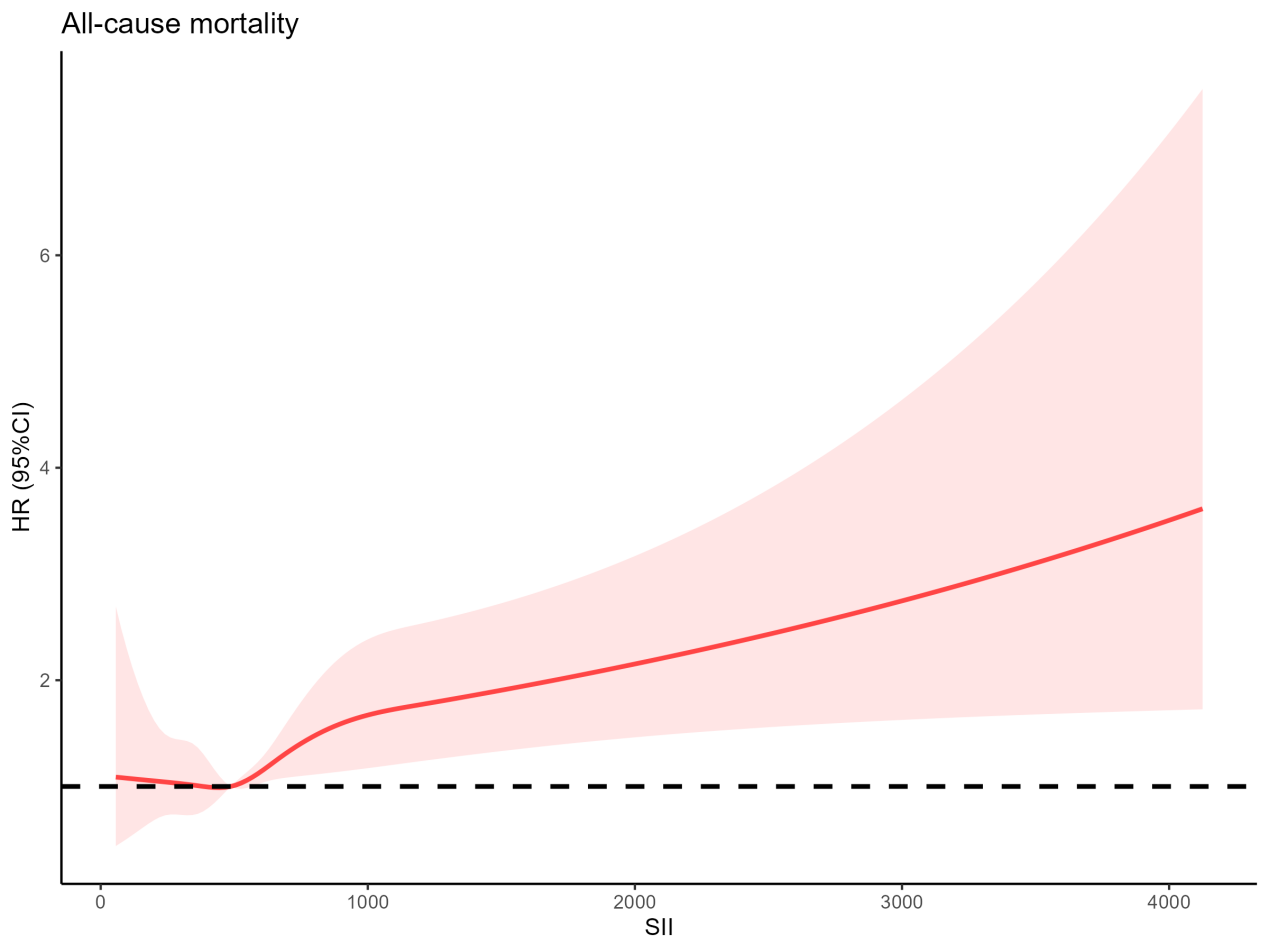


**Supplementary Figure S4.** RCS curve Association of SII Levels With All-Cause Mortality Among Adults With T2D excluding Less Than 2 Years of Follow-up.Hazard ratios (solid lines) and 95% CIs (shaded areas) were adjusted for educational level (less than high school, High School Grad/GED or Equivalent, more than college, family income to poverty ratio (<1.0, 1.0-3.0, or ≥3.0), BMI (<25.0, 25.0-29.9, or ≥30.0), drinking status (nondrinker, moderate, or heavy), physical activity (inactive or active), smoking status (never smoker, former smoker, or current smoker), HbA1c level (<7.0% or ≥7.0%), HEI 2015 (in quartile), diagnosed cardiovascular disease (CVD), hyperlipidemia, self-reported hypertension, and diabetes medication use (none, oral glucose-lowering medication, only insulin, oral glucose lowering medication and insulin).


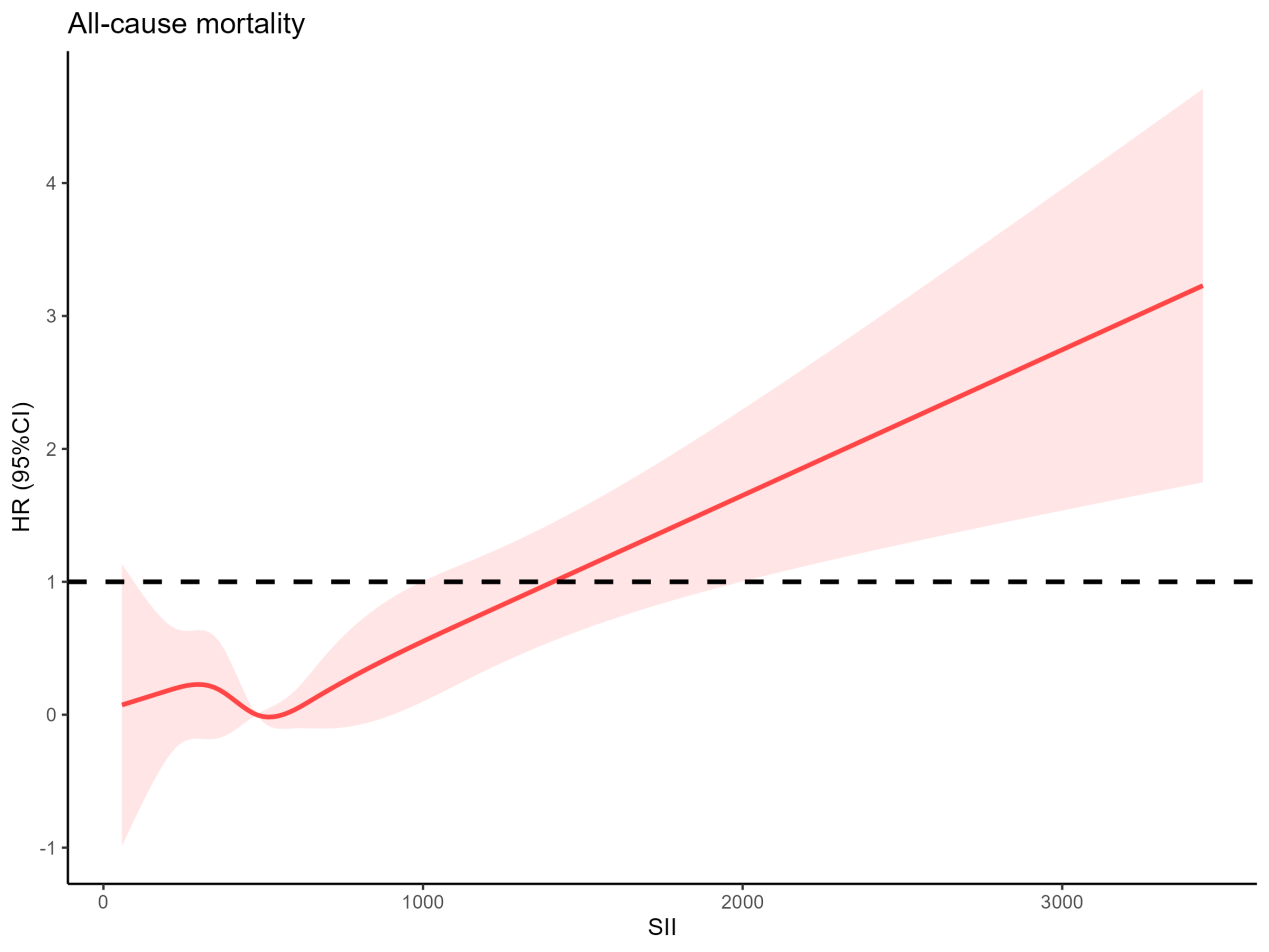


**Supplementary Figure S5.** RCS curve Association of SII Levels With All-Cause Mortality Among Adults With T2D excluding a CVD history at the baseline.Hazard ratios (solid lines) and 95% CIs (shaded areas) were adjusted for educational level (less than high school, High School Grad/GED or Equivalent, more than college, family income to poverty ratio (<1.0, 1.0-3.0, or ≥3.0), BMI (<25.0, 25.0-29.9, or ≥30.0), drinking status (nondrinker, moderate, or heavy), physical activity (inactive or active), smoking status (never smoker, former smoker, or current smoker), HbA1c level (<7.0% or ≥7.0%), HEI 2015 (in quartile), diagnosed cardiovascular disease (CVD), hyperlipidemia, self-reported hypertension, and diabetes medication use (none, oral glucose-lowering medication, only insulin, oral glucose lowering medication and insulin).


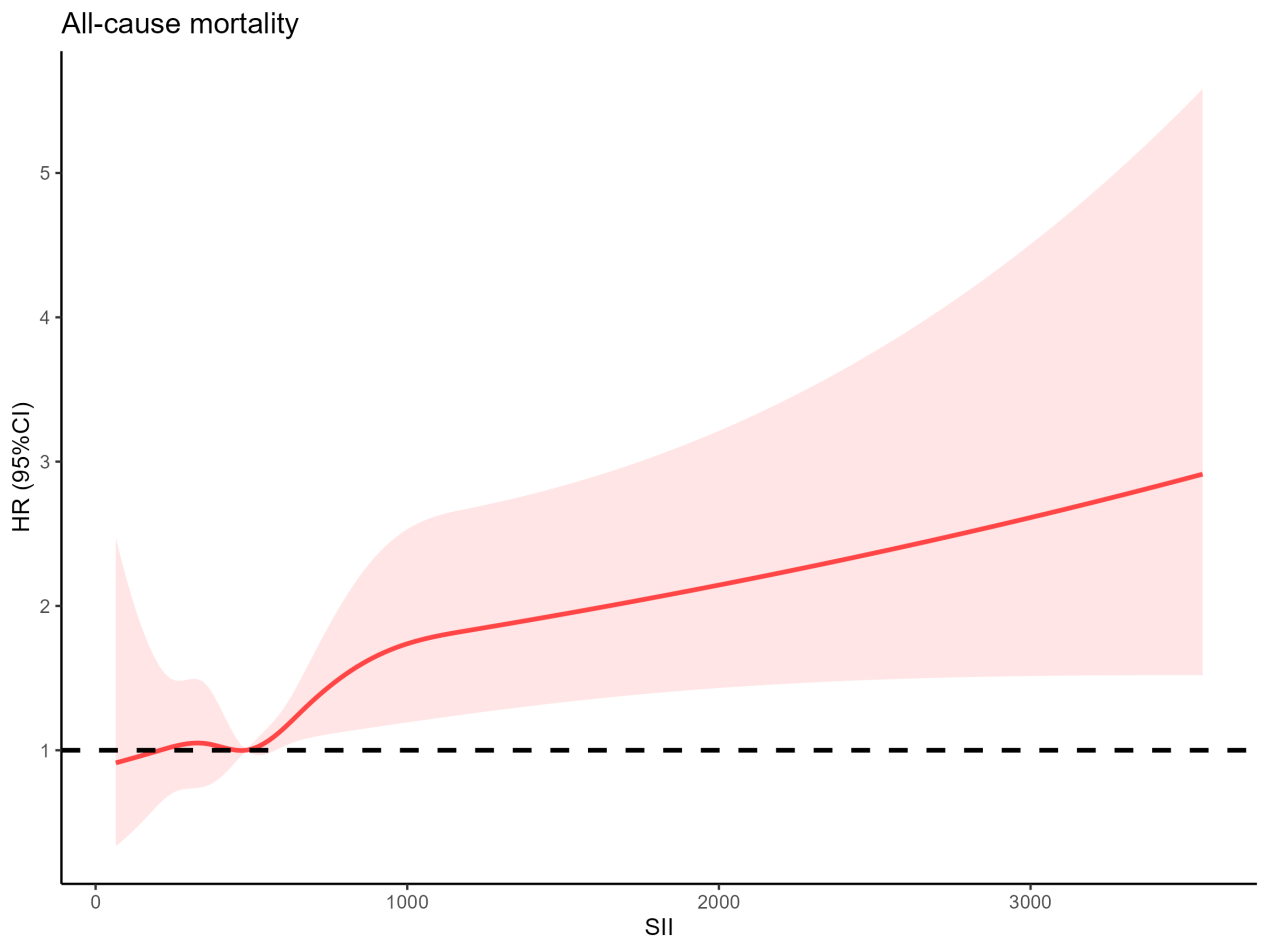


**Supplementary Figure S6.** RCS curve Association of SII Levels With All-Cause Mortality Among Adults With T2D excluding a cancer history at the baseline.Hazard ratios (solid lines) and 95% CIs (shaded areas) were adjusted for educational level (less than high school, High School Grad/GED or Equivalent, more than college, family income to poverty ratio (<1.0, 1.0-3.0, or ≥3.0), BMI (<25.0, 25.0-29.9, or ≥30.0), drinking status (nondrinker, moderate, or heavy), physical activity (inactive or active), smoking status (never smoker, former smoker, or current smoker), HbA1c level (<7.0% or ≥7.0%), HEI 2015 (in quartile), diagnosed cardiovascular disease (CVD), hyperlipidemia, self-reported hypertension, and diabetes medication use (none, oral glucose-lowering medication, only insulin, oral glucose lowering medication and insulin).


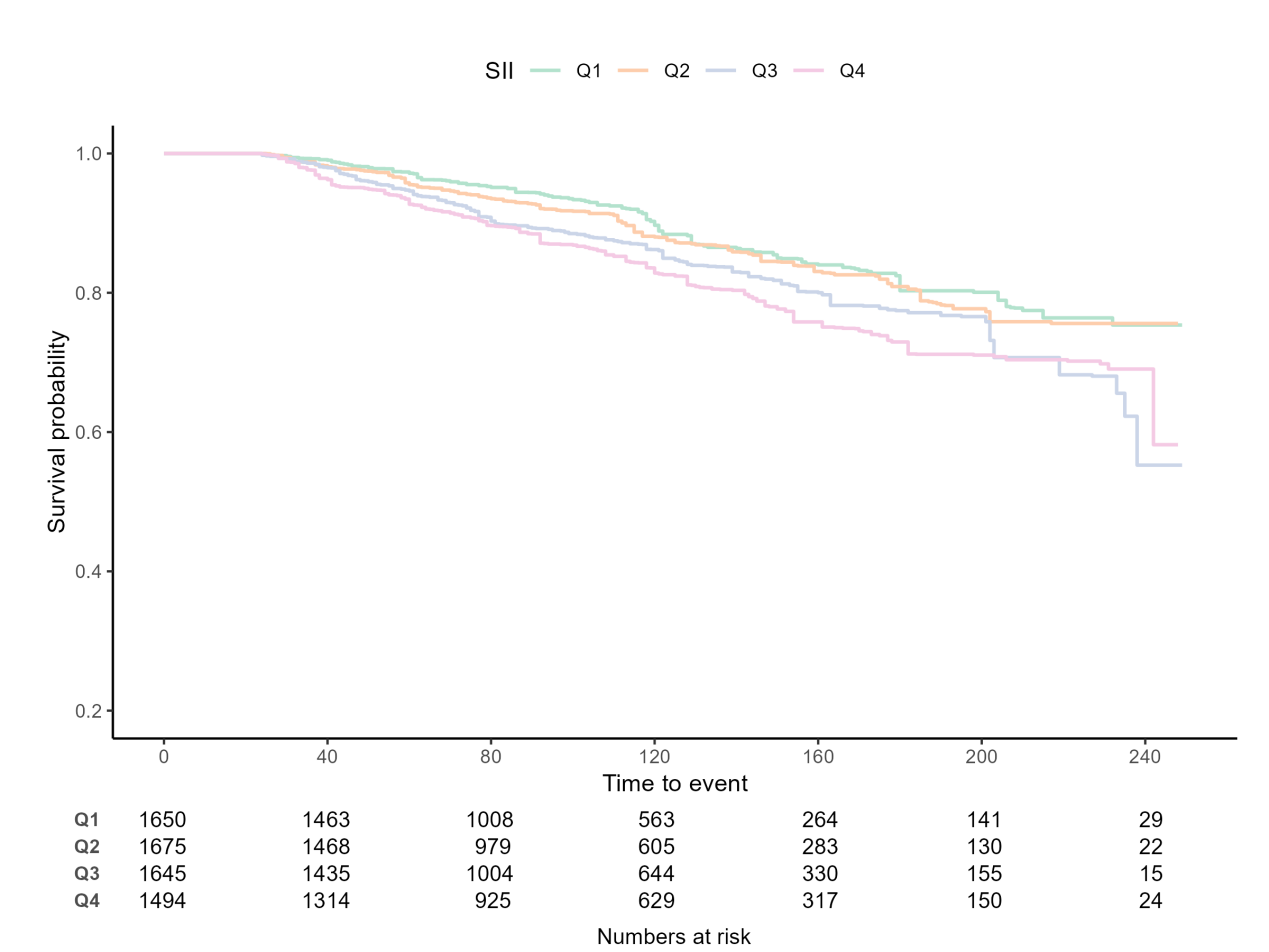


**Supplementary Figure S7.** Weighted Kaplan-Meier plots explaining the association of SII with Cardiovascular Mortality Among Adults With T2D excluding Less Than 2 Years of Follow-up.


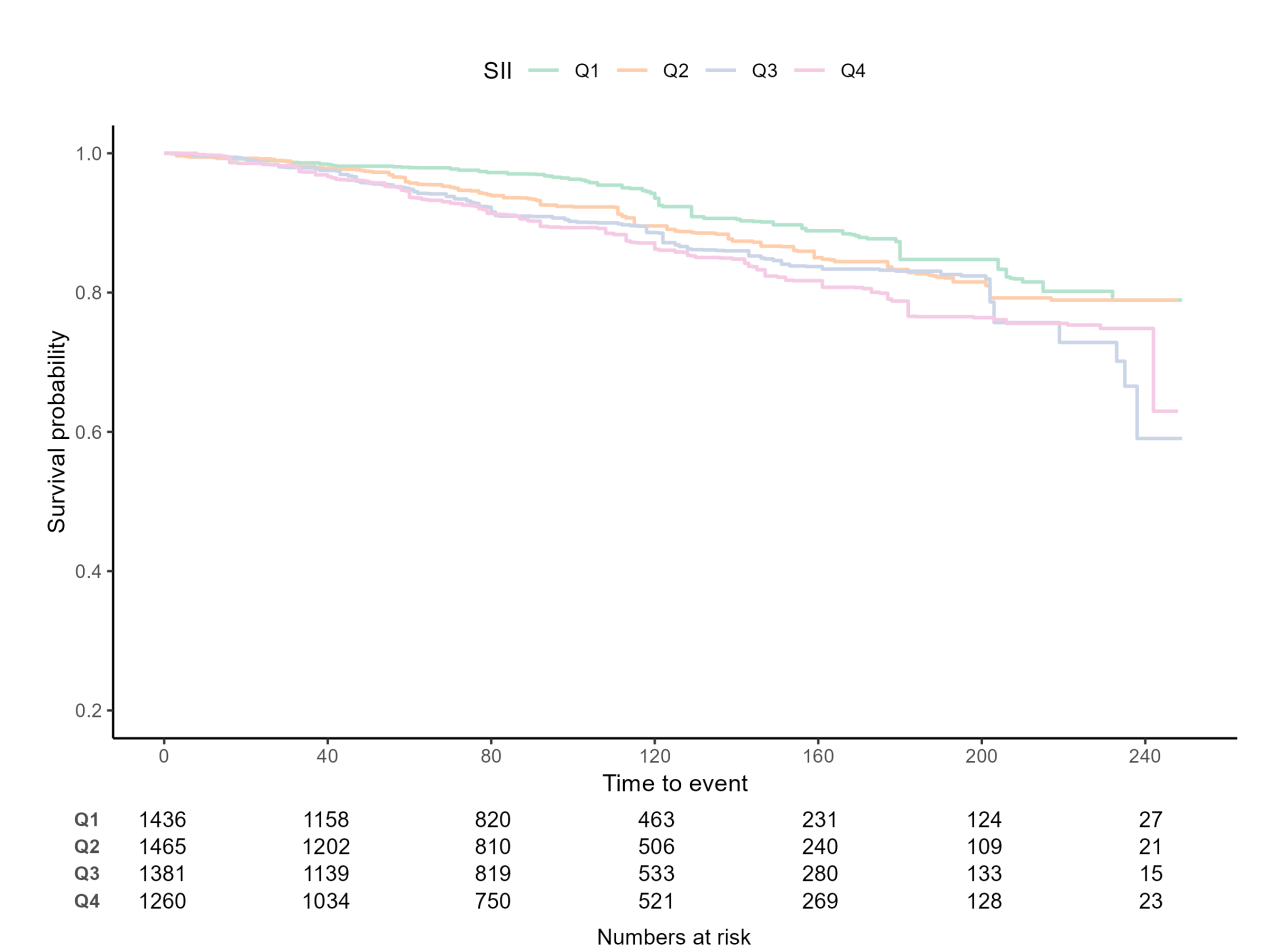


**Supplementary Figure S8.** Weighted Kaplan-Meier plots explaining the association of SII with Cardiovascular Mortality Among Adults With T2D excluding a CVD history at the baseline.


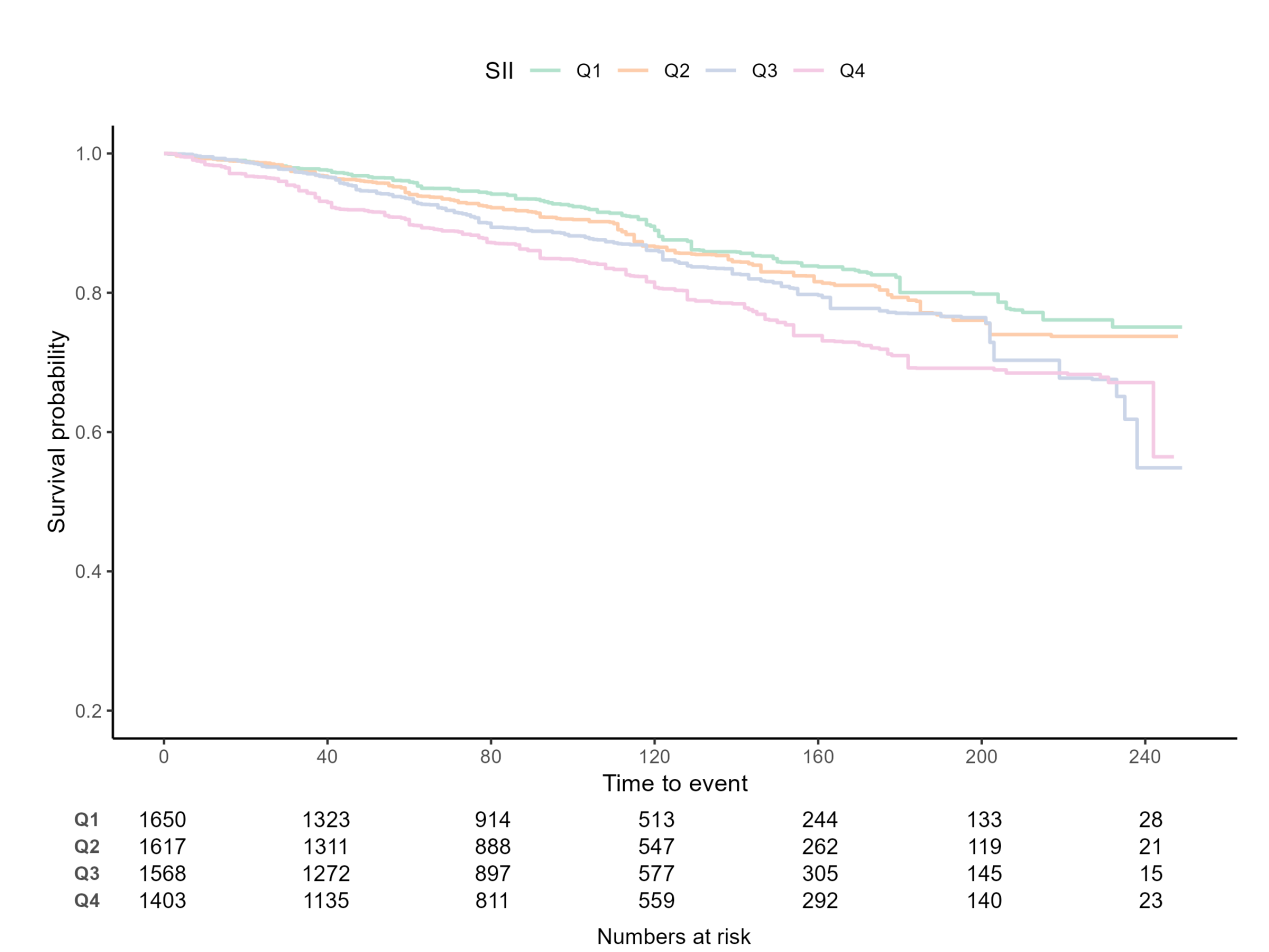


**Supplementary Figure S9.** Weighted Kaplan-Meier plots explaining the association of SII with Cardiovascular Mortality Among Adults With T2D excluding a cancer history at the baseline.

| Model | **Hazard ratio(95% CI) for Cardiovascular mortality** | | | |  |
| --- | --- | --- | --- | --- | --- |
|  | **Quartile of systemic immune-inflammtion index** | | | |  |
|  | Q1(17.3-345.0) | Q2(345.0-487.5) | Q3(487.5-702.6) | Q4(702.6-11700.0) | P_trend_ |
| Removing less than 24 months follow-up(N=6464) |  |  |  |  |  |
| Crude | 1(ref) | 1.18(0.88, 1.58) | 1.07(0.81, 1.41) | 1.47(1.10, 1.96) | 0.011 |
| Model 1 | 1(ref) | 1.21(0.94, 1.55) | 1.11(0.83, 1.48) | 1.58(0.70, 3.57) | <0.001 |
| Model 2 | 1(ref) | 1.55(0.82, 2.93) | 1.99(0.88, 4.50) | 1.7(1.26, 2.29) | 0.749 |
| Removing CVD at baseline(N=5542) |  |  |  |  |  |
| Crude | 1(ref) | 1.24(0.86, 1.78) | 1.13(0.82, 1.55) | 1.66(1.15, 2.42) | 0.03 |
| Model 1 | 1(ref) | 1.32(0.97, 1.80) | 1.18(0.83, 1.67) | 2.37(0.78, 7.21) | 0.123 |
| Model 2 | 1(ref) | 1.38(0.50, 3.81) | 1.42(0.55, 3.62) | 2.04(1.38, 3.03) | 0.312 |
| Removing cancer at baseline(N=6238) |  |  |  |  |  |
| Crude | 1(ref) | 1.38(0.50, 3.81) | 1.42(0.55, 3.62) | 2.37(0.78, 7.21) | 0.154 |
| Model 1 | 1(ref) | 1.07(0.84, 1.38) | 1(0.74, 1.35) | 1.46(0.65, 3.26) | 0.018 |
| Model 2 | 1(ref) | 1.29(0.62, 2.69) | 1.6(0.68, 3.79) | 1.59(1.17, 2.16) | 0.479 |

**Supplementary Table S2.** Hazard ratios of Cardiovascular Mortality by SII Levels Among Adults With T2D Excluding Less Than 2 Years of Follow-up, CVD and cancer at baseline. Hazard ratios (95% CI) were adjusted for educational level (less than high school, High School Grad/GED or Equivalent, more than college, family income to poverty ratio (<1.0, 1.0-3.0, or ≥3.0), BMI (<25.0, 25.0-29.9, or ≥30.0), drinking status (nondrinker, moderate, or heavy), physical activity (inactive or active), smoking status (never smoker, former smoker, or current smoker), HbA1c level (<7.0% or ≥7.0%), HEI 2015 (in quartile), diagnosed cardiovascular disease (CVD), hyperlipidemia, self-reported hypertension, and diabetes medication use (none, oral glucose-lowering medication, only insulin, oral glucose lowering medication and insulin).


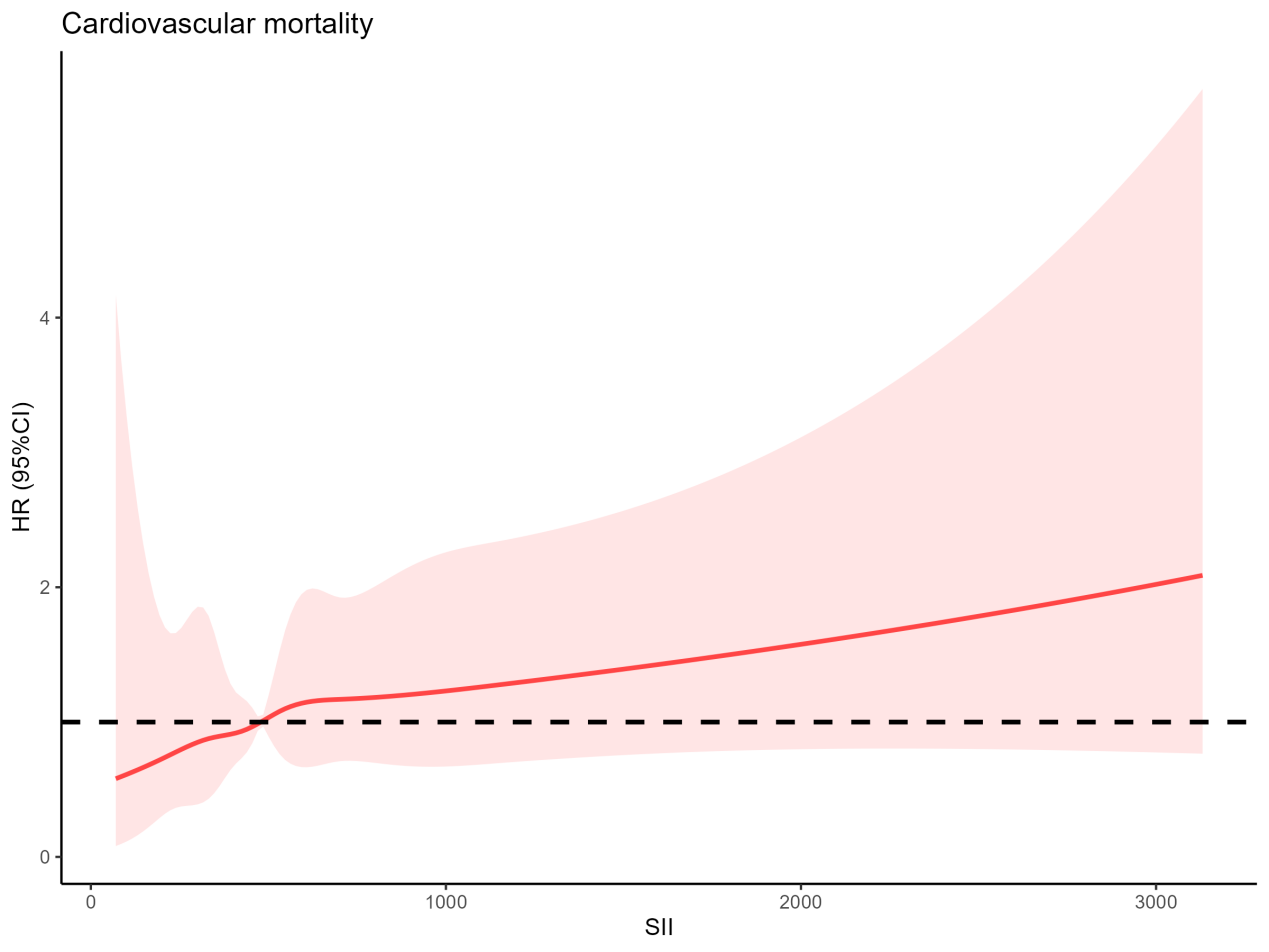


**Supplementary Figure S10.** RCS curve Association of SII Levels With Cardiovascular Mortality Among Adults With T2D excluding Less Than 2 Years of Follow-up.Hazard ratios (solid lines) and 95% CIs (shaded areas) were adjusted for educational level (less than high school, High School Grad/GED or Equivalent, more than college, family income to poverty ratio (<1.0, 1.0-3.0, or ≥3.0), BMI (<25.0, 25.0-29.9, or ≥30.0), drinking status (nondrinker, moderate, or heavy), physical activity (inactive or active), smoking status (never smoker, former smoker, or current smoker), HbA1c level (<7.0% or ≥7.0%), HEI 2015 (in quartile), diagnosed cardiovascular disease (CVD), hyperlipidemia, self-reported hypertension, and diabetes medication use (none, oral glucose-lowering medication, only insulin, oral glucose lowering medication and insulin).


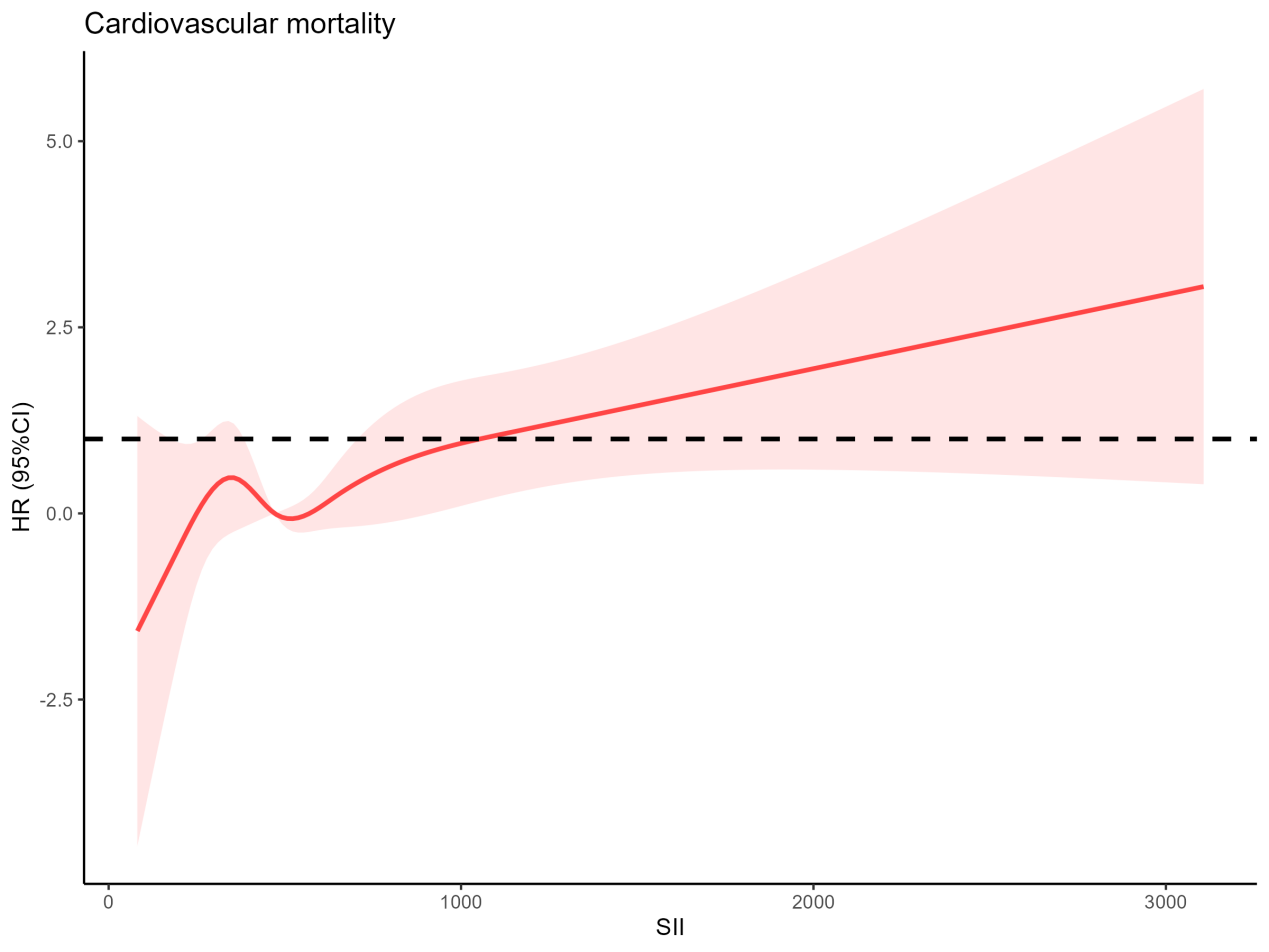


**Supplementary Figure S11.** RCS curve Association of SII Levels With Cardiovascular Mortality Among Adults With T2D excluding a CVD history at the baseline.Hazard ratios (solid lines) and 95% CIs (shaded areas) were adjusted for educational level (less than high school, High School Grad/GED or Equivalent, more than college, family income to poverty ratio (<1.0, 1.0-3.0, or ≥3.0), BMI (<25.0, 25.0-29.9, or ≥30.0), drinking status (nondrinker, moderate, or heavy), physical activity (inactive or active), smoking status (never smoker, former smoker, or current smoker), HbA1c level (<7.0% or ≥7.0%), HEI 2015 (in quartile), diagnosed cardiovascular disease (CVD), hyperlipidemia, self-reported hypertension, and diabetes medication use (none, oral glucose-lowering medication, only insulin, oral glucose lowering medication and insulin).


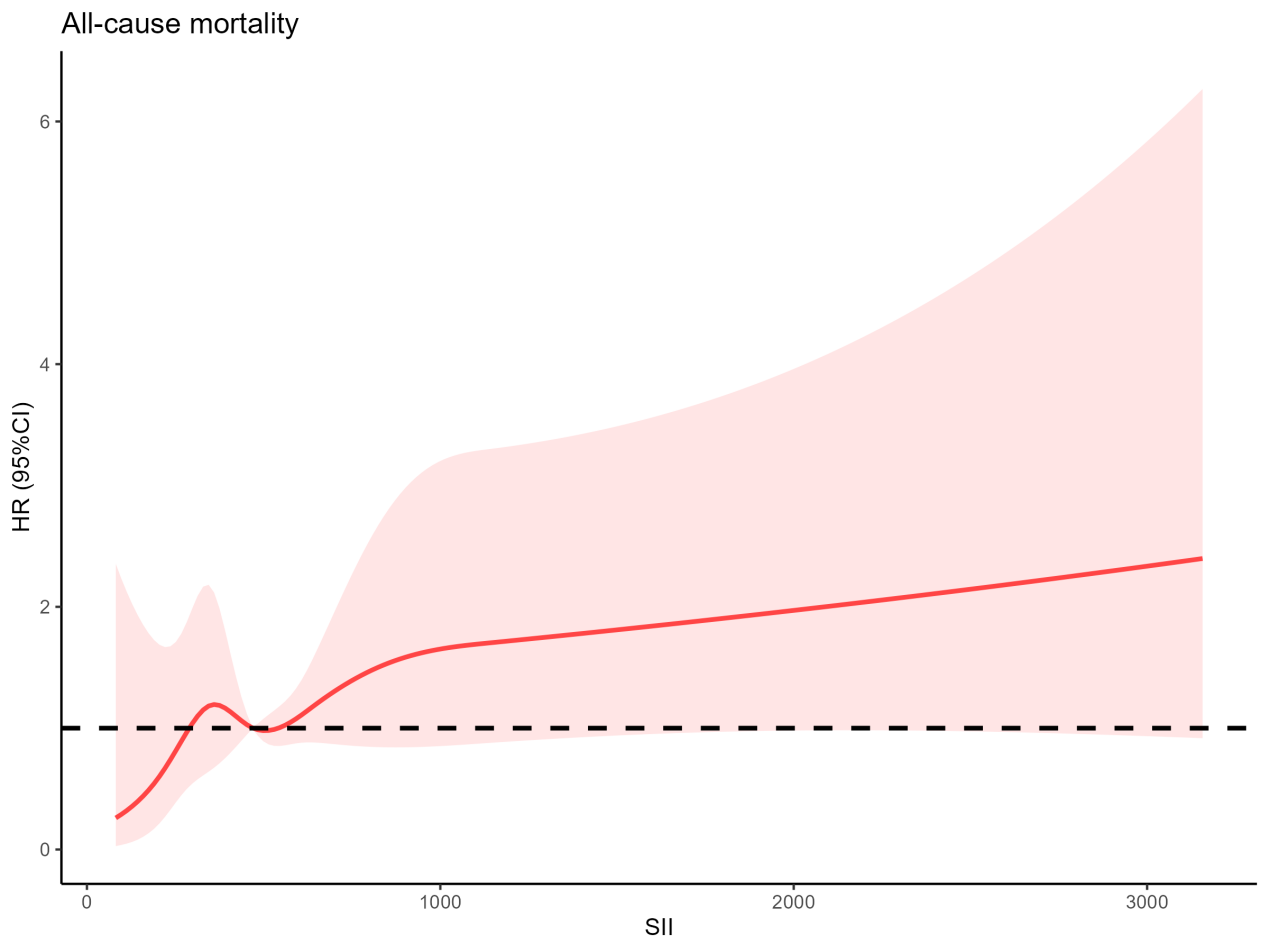


**Supplementary Figure S12.** RCS curve Association of SII Levels With Cardiovascular Mortality Among Adults With T2D excluding a cancer history at the baseline.Hazard ratios (solid lines) and 95% CIs (shaded areas) were adjusted for educational level (less than high school, High School Grad/GED or Equivalent, more than college, family income to poverty ratio (<1.0, 1.0-3.0, or ≥3.0), BMI (<25.0, 25.0-29.9, or ≥30.0), drinking status (nondrinker, moderate, or heavy), physical activity (inactive or active), smoking status (never smoker, former smoker, or current smoker), HbA1c level (<7.0% or ≥7.0%), HEI 2015 (in quartile), diagnosed cardiovascular disease (CVD), hyperlipidemia, self-reported hypertension, and diabetes medication use (none, oral glucose-lowering medication, only insulin, oral glucose lowering medication and insulin).
